# Synthesis of Formate Esters and Formamides Using an Au/TiO_2_-Catalyzed Aerobic Oxidative Coupling of Paraformaldehyde

**DOI:** 10.3390/nano7120440

**Published:** 2017-12-12

**Authors:** Ioannis Metaxas, Eleni Vasilikogiannaki, Manolis Stratakis

**Affiliations:** Department of Chemistry, University of Crete, Voutes, 71003 Iraklion, Greece; giannis.metaxas93@gmail.com (I.M.); vasilikelina@hotmail.com (E.V.)

**Keywords:** formates, formamides, paraformaldehyde, aerobic coupling, Au nanoparticles

## Abstract

A simple method for the synthesis of formate esters and formamides is presented based on the Au/TiO_2_-catalyzed aerobic oxidative coupling between alcohols or amines and formaldehyde. The suitable form of formaldehyde is paraformaldehyde, as cyclic trimeric 1,3,5-trioxane is inactive. The reaction proceeds via the formation of an intermediate hemiacetal or hemiaminal, respectively, followed by the Au nanoparticle-catalyzed aerobic oxidation of the intermediate. Typically, the oxidative coupling between formaldehyde (2 equiv) and amines occurs quantitatively at room temperature within 4 h, and there is no need to add a base as in analogous coupling reactions. The oxidative coupling between formaldehyde (typically 3 equiv) and alcohols is unprecedented and occurs more slowly, yet in good to excellent yields and selectivity. Minor side-products (2–12%) from the acetalization of formaldehyde by the alcohol are also formed. The catalyst is recyclable and can be reused after a simple filtration in five consecutive runs with a small loss of activity.

## 1. Introduction

Supported Au nanoparticles (Au NPs) have emerged during the past two decades as potent catalysts in several organic transformations. The surprising initial observation by Haruta’s group, that Au NPs are capable of oxidizing CO into CO_2_ even at −70 °C by atmospheric air [1,2], a reaction with great environmental significance, was the starting point of the extended growth of the applications of Au NPs in catalysis. This first breakthrough observation established a metal (Au) that no one could foresee having any major catalytic activity, into the first row of exploration by many research groups worldwide.

The reason for this catalytic activity is that the small clusters of Au deposited on several supports, such as metal oxides or polymeric materials, in contrast to bulk gold, which is formally inactive, exhibit unexpected properties. The key role of the support is not only to stabilize and protect Au clusters from leaking, aggregation, or against oxidation, which deactivates them in certain cases, but, in many reports, it is proposed that there is a synergy in the catalytic activity among metal oxide and Au NPs.

Following the Au NPs-catalyzed aerobic oxidation of CO, the first major step towards their recognition as powerful catalysts was triggered by their application in the aerobic oxidation of organic compounds, such as alcohols, aldehydes, amines, alkenes, etc. [3,4,5,6,7,8]. Such transformations gained significant attention within the organic community, due to their green nature, as the oxidant is simply the atmospheric air. The second major step in the field of catalysis using Au NPs, which essentially expanded during the first decade of this century, was rather unexpected, as nanogold catalysts found catalytic applications in organic transformations beyond aerobic oxidations. Such transformations are typically catalyzed either by ionic Au substances or by Pd(0) and other noble metals, mostly under homogeneous conditions [9,10,11,12,13].

**Scheme 1:**
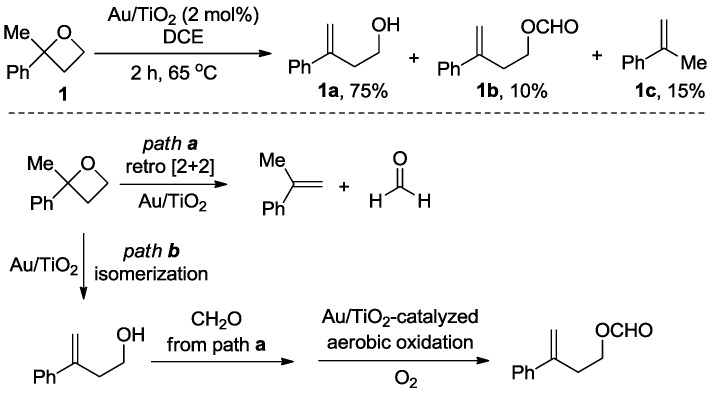
The transformations of an oxetane (**1**) in the presence of Au/TiO_2_ and a proposed mechanism, which triggered the current study of a methodology for the formation of formate esters and formamides via the aerobic oxidative coupling between alcohols/amines and paraformaldehyde.

The results that will be presented and analyzed below were triggered during our recent studies on the difunctionalization of oxetanes with a silylborane catalyzed by supported Au nanoparticles [14]. It was found that activated α,α-disubstituted oxetanes do not undergo silaboration and, in the absence of the silylborane, two competing Au-nanoparticle-catalyzed pathways take place in 1,2-dichloroethane (DCE) as a solvent: isomerization into homoallylic alcohols or a retro [2+2] decomposition forming an alkene and formaldehyde. Generally, the first path is prevailing. A typical example is shown in Scheme 1, regarding the case of 2-methyl-2-phenyloxetane (**1**).

Surprisingly, though, one of the minor side-products was the formate of the homoallylic alcohol (**1b**), and we reasonably considered its formation as arising from the reaction of homoallylic alcohol **1a** (isomerization product of the main path **a**) with formaldehyde, which is formed via the retro [2+2] decomposition path of the oxetane (path **b**). The isomerization mechanism of activated oxetanes into homoallylic alcohols catalyzed by Au/TiO_2_ (path **b**) is obviously identical to the concerted process for the isomerization of activated epoxides into allylic alcohols, previously established by our group [15] and requires the synergy among electrophilic Au nanoparticles and the basic oxygen atoms of the support. Additional experiments established that, under the reaction conditions, alkene **1c** does not arise via a retro carbonyl-ene reaction of homoallylic alcohol **1a**, but also, **1a** is not formed via a direct carbonyl-ene reaction among alkene **1c** and formaldehyde.

We, therefore, focused on a possible methodology for the synthesis of formate esters, based on the reaction between an alcohol and formaldehyde in the presence of Au/TiO_2_ under aerobic conditions. In this concept, essentially the in situ catalytic oxidation of the produced labile hemiacetal is anticipated. As a second task, we extended this concept to the mild and facile synthesis of formamides from the corresponding amines without using an external base as in previous reported analogous oxidative coupling protocols.

## 2. Results

### 2.1. Synthesis of Formate Esters

**Scheme 2:**
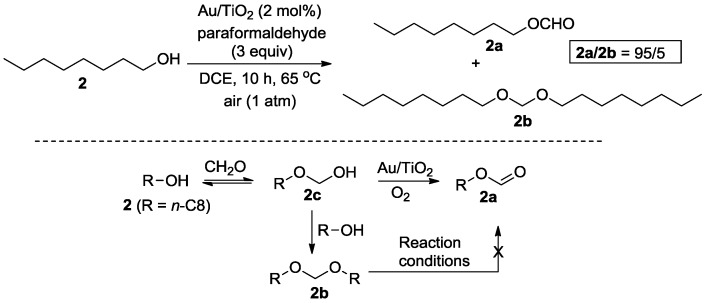
Aerobic oxidative coupling between 1-octanol and paraformaldehyde forming 1-octyl formate (**2b**) catalyzed by Au/TiO_2_ and the proposed mechanism.

To test this concept, we initially examined the reaction of a simple alcohol (1-octanol, **2**) with formaldehyde in the presence of Au/TiO_2_ (2 mol %). There are two stable solid sources of formaldehyde: paraformaldehyde, which is a linear polymeric form and delivers formaldehyde upon heating, and 1,3,5-trioxane, which is the cyclic trimeric acetal form. Treatment of 1-octanol with 3 molar equivalents of paraformaldehyde in 1,2-dichloroethane (DCE) and heating to 65 °C for 10 h, led to the quantitative consumption of the alcohol and to a mixture of formate ester **2a** and acetal **2b** in a relative ratio **2a**/**2b** = 95/5 (Scheme 2). The mixture was chromatographed and **2a** was isolated in 80% yield. Contrary to paraformaldehyde, we could not achieve any reaction with 1,3,5-trioxane.

Acetal **2b** [16] is not a precursor of formate **2a**, as it was separately treated with paraformaldehyde under conditions identical to the reaction, but no reaction was seen. Apparently, from the reaction between 1-octanol and formaldehyde, the labile hemiacetal **2c** is reversibly formed, which then undergoes aerobic oxidation catalyzed by Au/TiO_2_, leading to formate **2a**. The aldehyde functionality of formate ester is deactivated toward further nucleophilic attack by an alcohol molecule, as the corresponding dioctyl carbonate ester was not detected by GC-MS.

For the optimization of the reaction, a series of solvents and Au nanoparticle catalysts [17] was tested. Au/TiO_2_ is more efficient compared to Au/Al_2_O_3_ or Au/ZnO. These commercially available catalysts have similar wt % content in Au and average nanoparticle size. As per the solvents, although in toluene the reaction is taking place slightly slowly, the product selectivity (**2a**/**2b**) is better than in DCE. All other solvents gave inferior results in terms of conversion rates or selectivity (Table 1). The catalyst is easily recyclable and reusable after filtration and drying in the oven at 90 °C for 2 h, in five consecutive runs (see footnote of Table 1). A small decline in activity was observed, apparently due to some loss of material during the recycling process.

**Figure 1:**
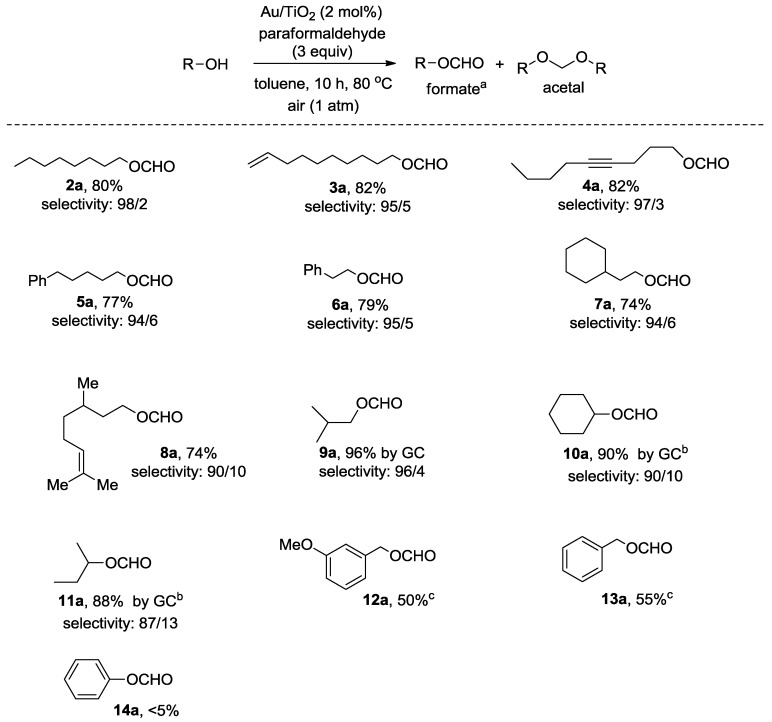
Formylation of alcohols via their aerobic oxidative coupling with paraformaldehyde catalyzed by Au/TiO_2_. ^a^ Isolated yields after column chromatography, except if referred as yields by GC. ^b^ Reaction time 18–22 h, 5 equiv of paraformaldehyde. ^c^ Side products from the partial oxidation of benzylic alcohols (~15%), acetalization of formaldehyde (~5–10%), and the formation of minor amounts of dibenzyl ethers (~10%) drops the yield of formates.

Subsequently, we tested a series of alcohols to examine the scope and limitations of their formylation, using Au/TiO_2_ (2 mol %) as the catalyst, 3 equiv of paraformaldehyde, toluene as the solvent, and heating to 80 °C for 10 h (Figure 1). Primary alcohols are, in general, good substrates and provide the corresponding formate esters in selectivity 92–99%. Primary benzylic alcohols are competitively oxidized into the corresponding benzaldehydes in a relative ratio of ~15–20%, while the formation of other minor side products drops the yield of formates to around 50–55%. Secondary alcohols are less prone to react due to steric reasons and require 5 equiv of paraformaldehyde and ~20 h to reach completion. Phenols are rather unreactive even using a large excess of paraformaldehyde (10 equiv) and extended reaction times; under these conditions <5% of formate ester was seen by GC-MS.

The Au-NP-catalyzed aerobic oxidation of hemiacetals has been well documented in the literature, as a protocol to prepare esters or lactones [18,19,20,21,22]. Yet for acyclic esters, one of the reactants has to be used in large excess (e.g., the reaction of benzaldehyde in methanol as a solvent to form methyl benzoate). Our approach is essentially the first example for the synthesis of formate esters employing a formaldehyde-alcohol oxidative coupling. Generally, formate esters can be prepared by the acid-catalyzed [23] or *N*-heterocyclic carbene-promoted [24] transesterification of alcohols with ethyl formate, and in the presence of PPh_3_/CBr_4_ [25], by the direct esterification of alcohols with formic acid catalyzed by I_2_ [26], and via the Pd-catalyzed carbonylation of alcohols with CO [27]. The only example for the synthesis of formate esters using Au nanoparticle catalysis, was recently reported by the groups of Hammond and Xu [28] employing a tandem Au/TiO_2_-catalyzed reduction/formylation of aromatic aldehydes with HCOOH.

### 2.2. Synthesis of Formamides

**Figure 2:**
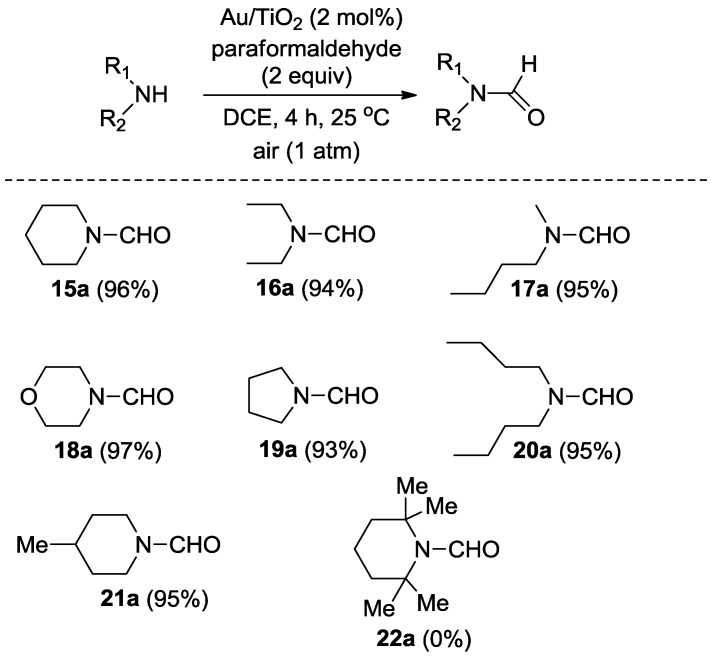
*N*-Formylation of secondary amines via oxidative coupling with paraformaldehyde catalyzed by Au/TiO_2_.

Given the ability of alcohols to oxidatively couple with paraformaldehyde yielding formate esters, we decided to examine the analogous coupling reaction using amines. To our delight, a series of secondary amines couple in the presence of Au/TiO_2_ (2 mol %) quantitatively with paraformaldehyde (2.0 equiv) in DCE as a solvent to form cleanly the corresponding formamides within 4 h at room temperature (Figure 2). The resulting products are pure and there is no need for chromatographic purification. An exception was the bulky 2,2,6,6-tetramethylpiperidine (**22**) which did not provide any product. The reaction was performed on 1.0 gr scale of piperidine, and reached completion after 32 h in ambient conditions, yielding formamide **15a** in 86% isolated yield. The extended reaction time is due to the fact that both small- and large-scale reactions are run into the same flask and the limiting factor is the amount of molecular oxygen at the liquid-air interface. From the mechanistic point of view, amine attacks formaldehyde, forming the corresponding hemiaminal, which is fast and cleanly oxidized by O_2_ into the corresponding formamides, a process catalyzed by Au nanoparticles.

There are a few recent analogous Au-catalyzed reports on the synthesis of formamides utilizing the formaldehyde/amine oxidative coupling concept. Shi and colleagues [29] have used a homemade Au/Al_2_O_3_ catalyst, which operates at room temperature and 1 atm O_2_ pressure and requires a base, such as NaOH in H_2_O as a solvent. Doris and Namboothiri [30] used a carbon nanotube-Au nanohybrid as a catalyst, and aqueous toluene as a solvent, while NaOH is also required. Prior to these reports, Sakurai’s group had shown an analogous NaOH-promoted coupling using Au nanoclusters stabilized by poly(*N*-vinyl-2-pyrrolidone) as a catalyst [31]. The characteristics of our catalytic system are that it is commercially available, provides quantitative reactions within a relatively short period of time (as all previous protocols more or less do), it simply operates under an open-air flask, while there is no need of a base, as in all analogous oxidative coupling protocols reported to date.

It is possible that the support of Au nanoparticles (titania) itself plays the role of a base in our process. We had previously invoked the crucial role of oxygen atoms of TiO_2_ as the required basic site in the synergistic isomerization of epoxides into allylic alcohols catalyzed by Au/TiO_2_ [15]. Note that formamides can also be prepared via nano Au-catalyzed coupling reactions between amines and methanol [32,33]. In those protocols which require rather harsh conditions and excess of reagents, methanol is oxidized into formaldehyde, which then undergoes oxidative coupling with the amine. Surprisingly, in one of these reports [33], where Au-nanopore was used as a catalyst, the direct oxidative coupling between formaldehyde and an amine was unsuccessful. The concept of coupling alcohols other than methanol, and amines for the synthesis of amides under Au nanoparticle-catalysis, has also been shown to be synthetically useful [34,35]. Finally, Au nanoparticle catalysis has also been used in the synthesis of formamides by other approaches, such as in the reaction of amines with CO_2_ or ammonium formate under reductive conditions [36,37].

Apart from nano Au-catalysis, the synthesis of formamides is feasible in the literature based on the catalytic transamidation of amines with HCONH_2_ [38], treatment of amines with CO_2_ and a hydrosilane or with H_2_ under various catalytic conditions [39,40,41], Ru-catalyzed dehydrogenative coupling between amines [42] or nitriles [43] and methanol, and the base-catalyzed carbonylation of amines with carbon monoxide [44].

## 3. Experimental Section

### 3.1. Catalysts

The supported Au nanoparticles on TiO_2_, Al_2_O_3_, or ZnO having a gold content ~1 wt %, which were used in our studies, are commercially available from Strem Chemicals Inc. The average crystallite size of Au nanoparticles is ~2–3 nm. Prior to use, they were ground in a mortar, and the resulting purple dust was kept in a dark-colored bottle.

### 3.2. Catalytic Reactions

Typical reaction of alcohol or amine formylation: In a vial are placed 0.4 mmol of the alcohol, 1.2 mmol of paraformaldehyde, and 160 mg of Au/TiO_2_ (~1.0 mol % in Au, 2 mol %) in 2 mL of toluene. The heterogeneous mixture is heated to 80 °C until the alcohol is consumed. The slurry is then filtered with the aid of 2 mL of dichloromethane under a low pressure through a short pad of silica gel or celite, and the filtrate is evaporated. Chromatographic purification is accompanied, if required. Regarding the formylation of amines, the amount of paraformaldehyde used is 2.0 equivalents relative to the amine and the reaction takes place at room temperature. After filtration and evaporation of the solvents, the resulting formamides are pure and generally do not require any chromatographic purification.

### 3.3. Characterization of Products

*Octyl formate* (**2a**): ^1^H NMR (500 MHz, CDCl_3_): 8.06 (br s, 1H), 4.16 (td, *J*_1_ = 7.0 Hz, *J*_2_ = 0.5 Hz, 2H), 1.69–1.63 (m, 2H), 1.40–1.22 (m, 10H), 0.88 (t, *J* = 7.0 Hz, 3H); ^13^C NMR (125 MHz, CDCl_3_): 161.2, 64.1, 31.7, 29.1, 28.5, 25.8, 22.6, 14.1.

*Dec-9-en-1-yl formate* (**3a**): ^1^H NMR (500 MHz, CDCl_3_): 8.05 (br s, 1H), 5.80 (ddq, *J*_1_ = 16.0 Hz, *J*_2_ = 10.0 Hz, *J*_2_ = 7.0 Hz, 1H), 4.99 (br d, *J* = 16.0 Hz, 1H), 4.94 (br d, *J* = 10.0 Hz, 1H), 4.15 (td, *J*_1_ = 7.0 Hz, *J*_2_ = 0.5 Hz, 2H), 2.06–2.02 (m, 2H), 1.67–1.63 (m, 2H), 1.38–1.24 (m, 12H); ^13^C NMR (125 MHz, CDCl_3_): 161.2, 139.1, 114.1, 64.1, 33.8, 29.4, 29.3, 29.1, 29.0, 28.9, 28.5, 25.8.

*Dec-5-yn-1-yl formate* (**4a**): ^1^H NMR (500 MHz, CDCl_3_): 8.06 (br s, 1H), 4.18 (t, *J* = 7.0 Hz, 2H), 2.21–2.12 (m, 4H), 1.81–1.76 (m, 2H), 1.58–1.38 (m, 6H), 0.90 (t, *J* = 7.0 Hz, 3H); ^13^C NMR (125 MHz, CDCl_3_): 161.1, 80.9, 79.1, 63.6, 31.2, 27.6, 25.4, 21.9, 18.4, 18.3, 13.6.

*5-Phenylpentyl formate* (**5a**): ^1^H NMR (300 MHz, CDCl_3_): 8.06 (br s, 1H), 7.30–7.15 (m, 5H), 4.16 (t, *J* = 7.0 Hz, 2H), 2.61 (t, *J* = 7.5 Hz, 2H), 1.70–1.36 (m, 8H); ^13^C NMR (75 MHz, CDCl_3_): 161.2, 142.6, 128.4, 128.2, 125.6, 64.0, 35.8, 31.3, 28.8, 28.4, 25.7.

*Phenethyl formate* (**6a**): ^1^H NMR (300 MHz, CDCl_3_): 8.05 (br s, 1H), 7.36–7.23 (m, 5H), 4.40 (t, *J* = 7.0 Hz, 2H), 2.99 (t, *J* = 7.5 Hz, 2H); ^13^C NMR (75 MHz, CDCl_3_): 161.0, 137.4, 128.9, 128.6, 126.7, 64.4, 34.9.

*2-Cyclohexylethyl formate* (**7a**): ^1^H NMR (300 MHz, CDCl_3_): 8.06 (br s, 1H), 4.20 (t, *J* = 7.0 Hz, 2H), 1.75–0.88 (m, 13H); ^13^C NMR (75 MHz, CDCl_3_): 161.2, 62.2, 35.8, 34.4, 33.1, 26.4, 26.2.

*3,7-Dimethyloct-6-en-1-yl formate* (**8a**): ^1^H NMR (300 MHz, CDCl_3_): 8.06 (br s, 1H), 5.08 (t, *J* = 7.0 Hz, 2H), 4.23–4.16 (m, 2H), 2.05–1.93 (m, 2H), 1.72–1.15 (m, 7H), 1.71 (s, 3H), 1.65 (s, 3H), 0.92 (d, *J* = 7.0 Hz, 3H); ^13^C NMR (75 MHz, CDCl_3_): 161.2, 131.4, 124.4, 62.5, 36.9, 35.3, 29.3, 25.7, 25.3, 19.3, 17.6.

*3-Methoxybenzyl formate* (**12a**): ^1^H NMR (500 MHz, CDCl_3_): 8.15 (br s, 1H), 7.31–7.28 (m, 1H), 6.96–6.87 (m, 2H), 5.18 (s, 2H), 3.82 (s, 3H); ^13^C NMR (125 MHz, CDCl_3_): 160.7, 159.7, 136.6, 129.7, 120.4, 114.0, 113.6, 65.5, 55.2.

*Benzyl formate* (**13a**): ^1^H NMR (300 MHz, CDCl_3_): 8.15 (br s, 1H), 7.40–7.36 (m, 5H), 5.22 (br s, 2H); ^13^C NMR (75 MHz, CDCl_3_): 160.7, 135.2, 128.6, 128.5, 128.3, 65.7.

*Piperidine-1-carbaldehyde* (**15a**): ^1^H NMR (500 MHz, CDCl_3_): 7.99 (br s, 1H), 3.47 (t, *J* = 6.0 Hz, 2H), 3.29 (t, *J* = 6.0 Hz, 2H), 1.69–1.52 (m, 6H); ^13^C NMR (125 MHz, CDCl_3_): 160.8, 46.8, 40.6, 26.5, 25.0, 24.7.

*N,N-Diethylformamide* (**16a**): ^1^H NMR (500 MHz, CDCl_3_): 8.04 (br s, 1H), 3.36 (q, *J* = 7.5 Hz, 2H), 3.27 (q, *J* = 7.5 Hz, 2H), 1.18 (t, *J* = 7.5 Hz, 3H), 1.12 (t, *J* = 7.5 Hz, 3H); ^13^C NMR (125 MHz, CDCl_3_): 162.2, 41.8, 36.5, 14.8, 12.7.

*N-Butyl-N-methylformamide* (**17a**): ^1^H NMR (300 MHz, CDCl_3_); two rotamers in a relative ratio ~2/1: 8.04 (br s, 1H from the two rotamers), 3.29 (t, *J* = 7.0 Hz, 2H of the minor rotamer), 3.19 (t, *J* = 7.0 Hz, 2H of the major rotamer), 2.92 (s, 3H of the minor rotamer), 2.85 (s, 3H of the major rotamer), 1.70–1.52 (m, 2H from the two rotamers), 0.90 (t, *J* = 7.5 Hz, 3H of the minor rotamer), 0.89 (t, *J* = 7.5 Hz, 3H of the major rotamer); ^13^C NMR (75 MHz, CDCl_3_): 162.7 (major rotamer), 162.5 (minor rotamer), 51.2 (major rotamer), 45.7 (minor rotamer), 34.5 (minor rotamer), 29.3 (major rotamer), 21.1 (major rotamer), 19.9 (minor rotamer), 11.1 (minor rotamer), 10.7 (major rotamer).

*Morpholine-4-carbaldehyde* (**18a**): ^1^H NMR (500 MHz, CDCl_3_): 8.06 (br s, 1H), 3.70 (t, *J* = 5.0 Hz, 2H), 3.67 (t, *J* = 5.0 Hz, 2H), 3.58 (t, *J* = 5.0 Hz, 2H), 3.40 (t, *J* = 5.0 Hz, 2H); ^13^C NMR (125 MHz, CDCl_3_): 160.8, 67.2, 66.4, 45.8, 40.6.

*Pyrrolidine-1-carbaldehyde* (**19a**): ^1^H NMR (300 MHz, CDCl_3_): 8.30 (br s, 1H), 3.53 (t, *J* = 5.0 Hz, 2H), 3.44 (t, *J* = 5.0 Hz, 2H), 1.95–1.89 (m, 4H); ^13^C NMR (75 MHz, CDCl_3_): 161.0, 46.2, 43.3, 24.9, 24.2.

*N,N-Dibutylformamide* (**20a**): ^1^H NMR (500 MHz, CDCl_3_): 8.03 (br s, 1H), 3.27 (t, *J* = 7.5 Hz, 2H), 3.18 (q, *J* = 7.5 Hz, 2H), 1.54–1.46 (m, 4H), 1.34–1.26 (m, 4H), 0.92 (t, *J* = 7.5 Hz, 6H); ^13^C NMR (125 MHz, CDCl_3_): 162.7, 47.2, 41.8, 30.7, 29.6, 20.1, 19.6, 13.8, 13.6.

*4-Methylpiperidine-1-carbaldehyde* (**21a**): ^1^H NMR (300 MHz, CDCl_3_): 8.00 (br s, 1H), 4.38–4.32 (m, 1H), 3.60–3.54 (m, 1H), 3.09–2.99 (m, 1H), 2.65–2.55 (m, 1H), 1.77–1.52 (m, 3H), 1.18–1.02 (m, 2H), 0.95 (d, *J* = 6.5 Hz, 3H); ^13^C NMR (75 MHz, CDCl_3_): 160.8, 46.2, 39.9, 34.7, 33.2, 31.3, 21.7.

## 4. Conclusions

In conclusion, we present herein a novel method for the synthesis of formate esters through an aerobic oxidative coupling between alcohols and paraformaldehyde catalyzed by commercially available Au nanoparticles supported on TiO_2_. The coupling occurs through the Au nanoparticle-catalyzed oxidation of the labile hemiacetal by air. This concept was also applied to the synthesis of formamides. The formaldehyde-amine oxidative coupling occurs more readily than in the case of alcohols, and there is no need to use a base additive, as is the case in the currently known analogous coupling methodologies.

## Figures and Tables

**Table 1:** Optimization of conditions regarding the Au nanoparticle-catalyzed formylation of 1-octanol (**2**) with paraformaldehyde.

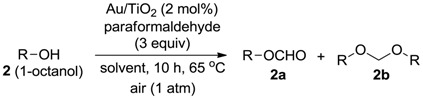				
**Entry**	**Catalyst**	**Solvent**	**Conversion (%)**	**Selectivity (2a/2b)**
1	Au/TiO_2_	1,2-Dichloroethane	100	95/5
2	Au/TiO_2_	Ethyl acetate	45	92/8
3	Au/TiO_2_	Tetrahydrofuran	34	79/21
4	Au/TiO_2_	Toluene	89 ^a,b^	98/2
5	Au/TiO_2_	Hexane	34	97/3
6	Au/Al_2_O_3_	1,2-Dichloroethane	35	96/4
7	Au/ZnO	1,2-Dichloroethane	50	98/2
8	Au/Al_2_O_3_	Toluene	5	Not Determined
9	Au/ZnO	Toluene	61	99/1

^a^ Conversion 100% after 12–13 h, or after 10 h at 80 °C. ^b^ Regarding recycling studies, in the 2nd run: 100% conversion after 13 h; 3rd run: 100% conversion after 15 h; 4th run: 100% conversion after 15 h; 5th run: 100% conversion after 16 h.

## Supplementary Materials

The ^1^H and ^13^C NMR copies of products are available online at http://www.mdpi.com/2079-4991/7/12/440/s1.
