# Supplementary material for: Synthesis of Formate Esters and Formamides Using an Au/TiO2-Catalyzed Aerobic Oxidative Coupling of Paraformaldehyde
Source: Nanomaterials (Basel). 2017 Dec 12;7(12):440. doi: 10.3390/nano7120440 (PMC5746930; doi:10.3390/nano7120440)
Supplement: Supplementary file 1 [file nanomaterials-07-00440-s001.pdf]

SUPPORTING INFORMATION

Synthesis of formate esters and formamides using a Au/TiO_2-catalyzed aerobic oxidative coupling of paraformaldehyde

Ioannis Metaxas, Eleni Vasilikogiannaki and Manolis Stratakis*

Department of Chemistry, University of Crete, Voutes 71003 Iraklion, Greece

stratakis@uoc.gr

^{1}H and ^{13}C NMR spectra

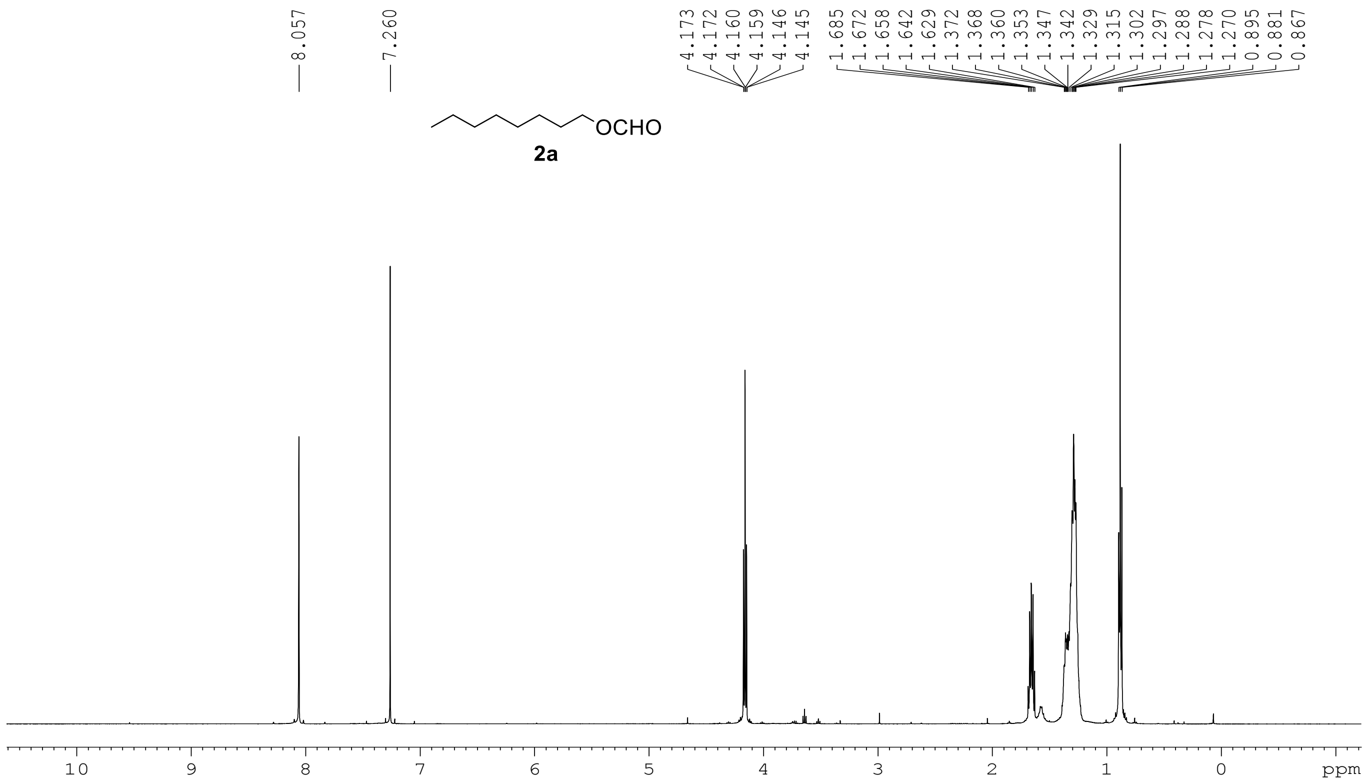

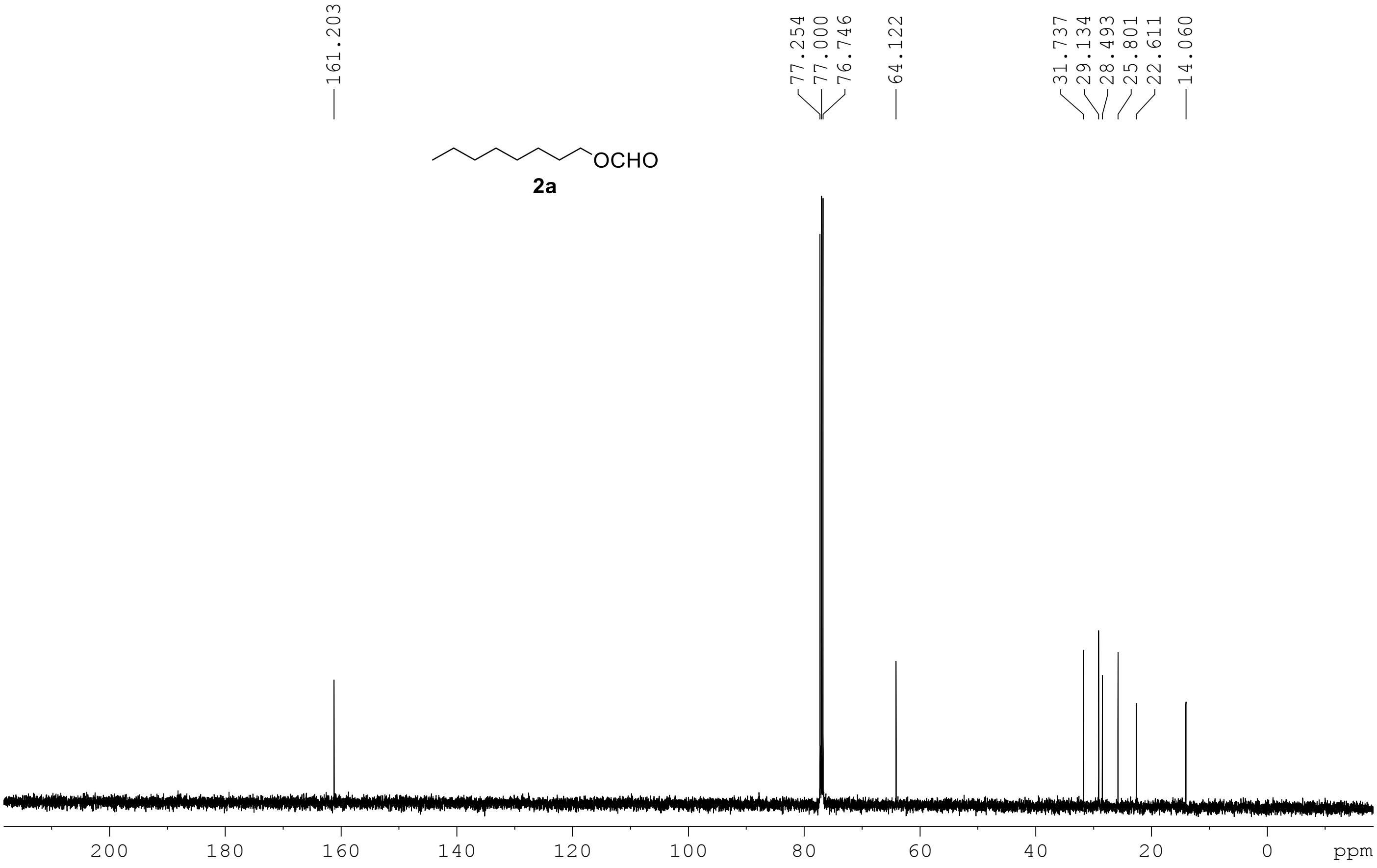

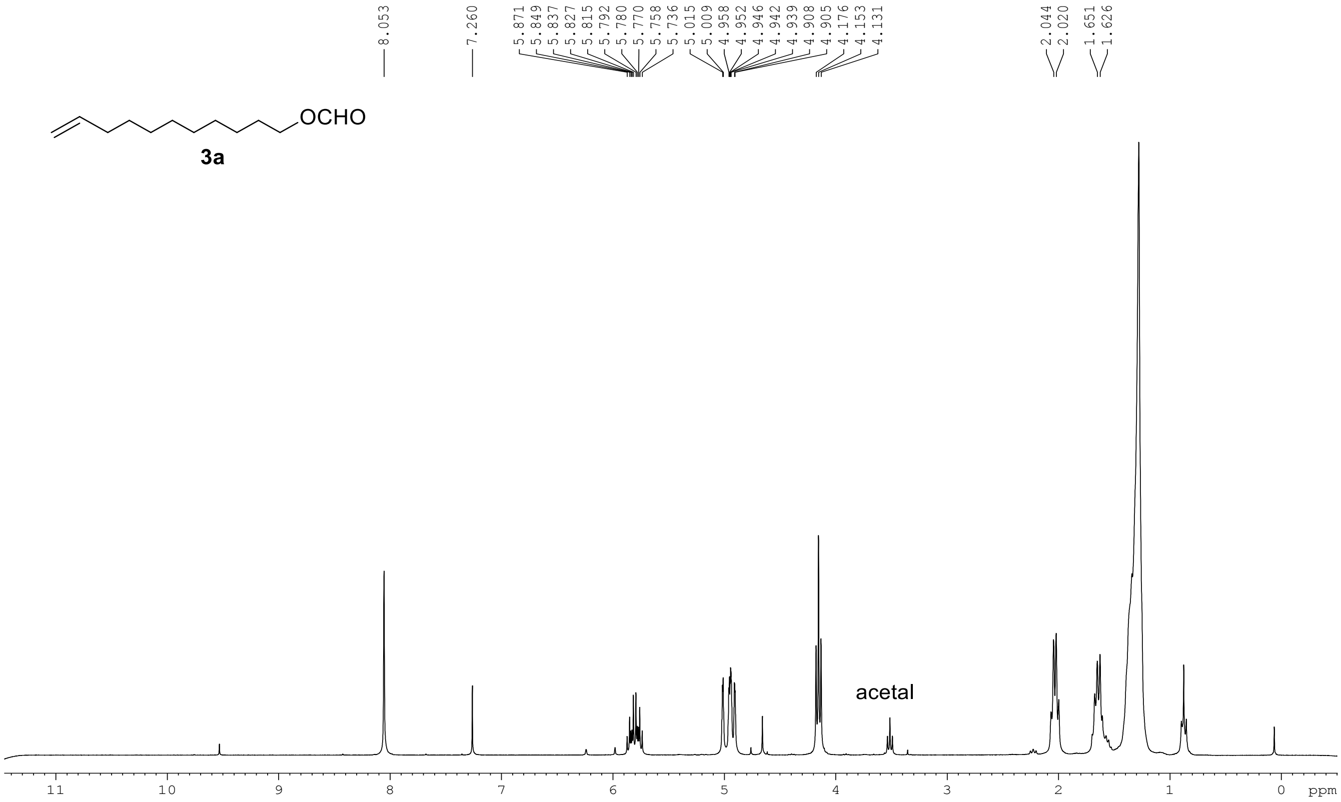
8.053
7.260
5.871 5.849 5.837 5.827 5.815 5.792 5.780 5.770 5.758 5.736 5.015 5.009 4.958 4.952 4.946 4.942 4.939 4.908 4.905 4.176 4.153 4.131
2.044 2.020 1.651 1.626
OCHO
3a
acetal
11 10 9 8 7 6 5 4 3 2 1 0 ppm

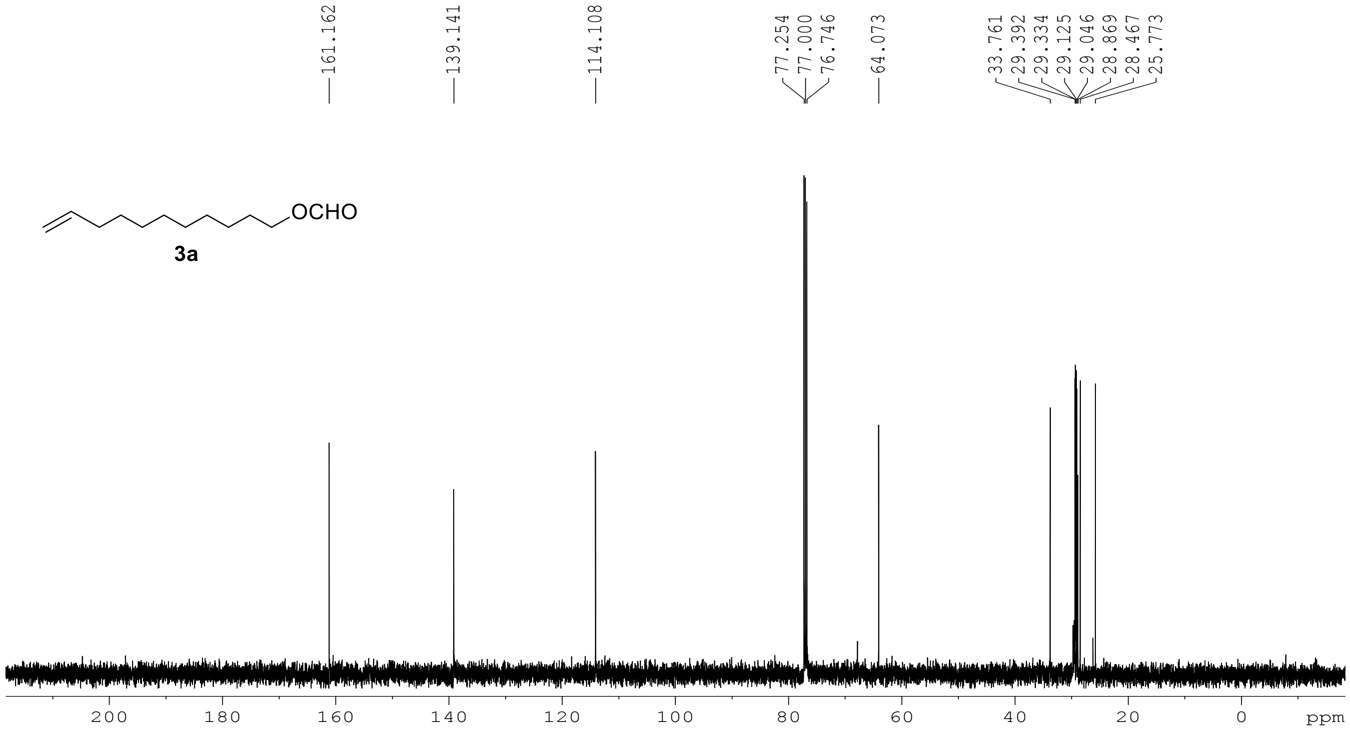
161.162
139.141
114.108
77.254 77.000 76.746
64.073
33.761 29.392 29.334 29.125 29.046 28.869 28.467 25.773
OCHO
3a
200 180 160 140 120 100 80 60 40 20 0 ppm

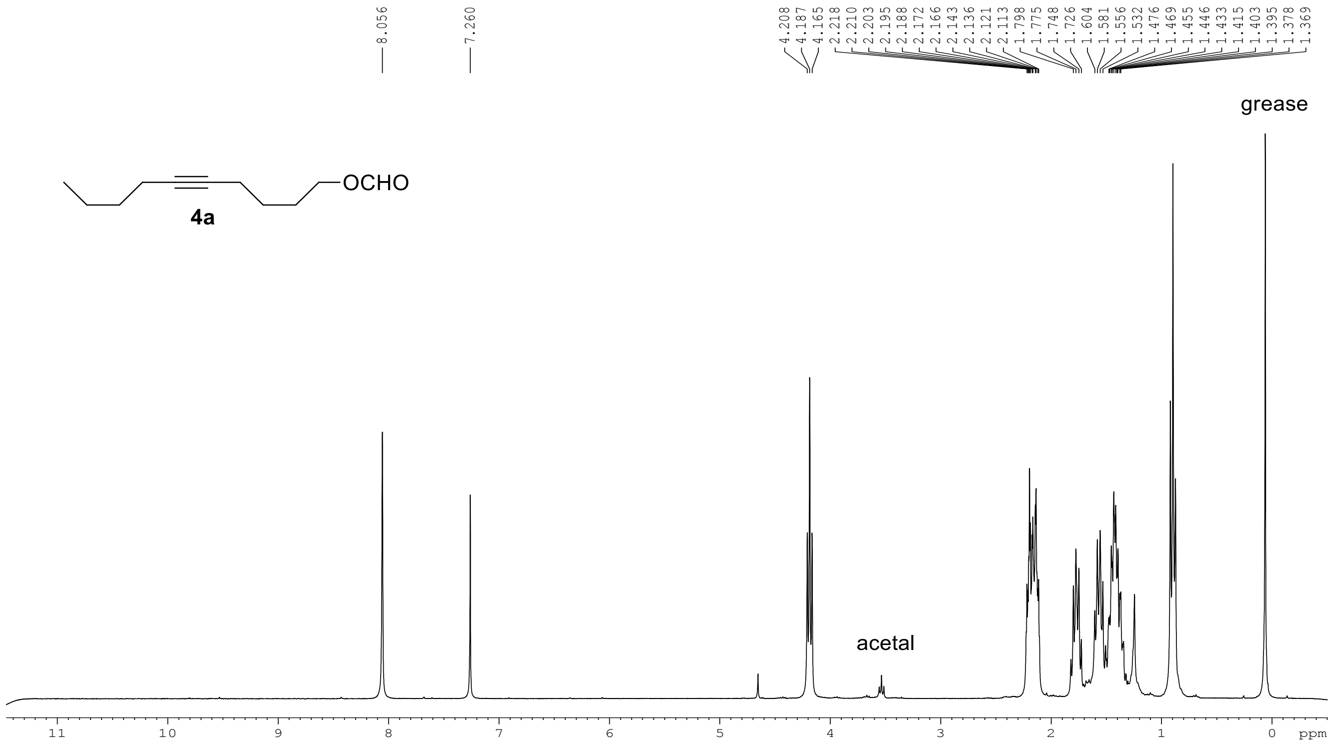
8.056
7.260
4.208
4.187
4.165
2.218
2.210
2.203
2.195
2.188
2.172
2.166
2.143
2.136
2.121
2.113
1.798
1.775
1.748
1.726
1.604
1.581
1.556
1.532
1.476
1.469
1.455
1.446
1.433
1.415
1.403
1.395
1.378
1.369
grease
OCHO
4a
acetal
11 10 9 8 7 6 5 4 3 2 1 0 ppm

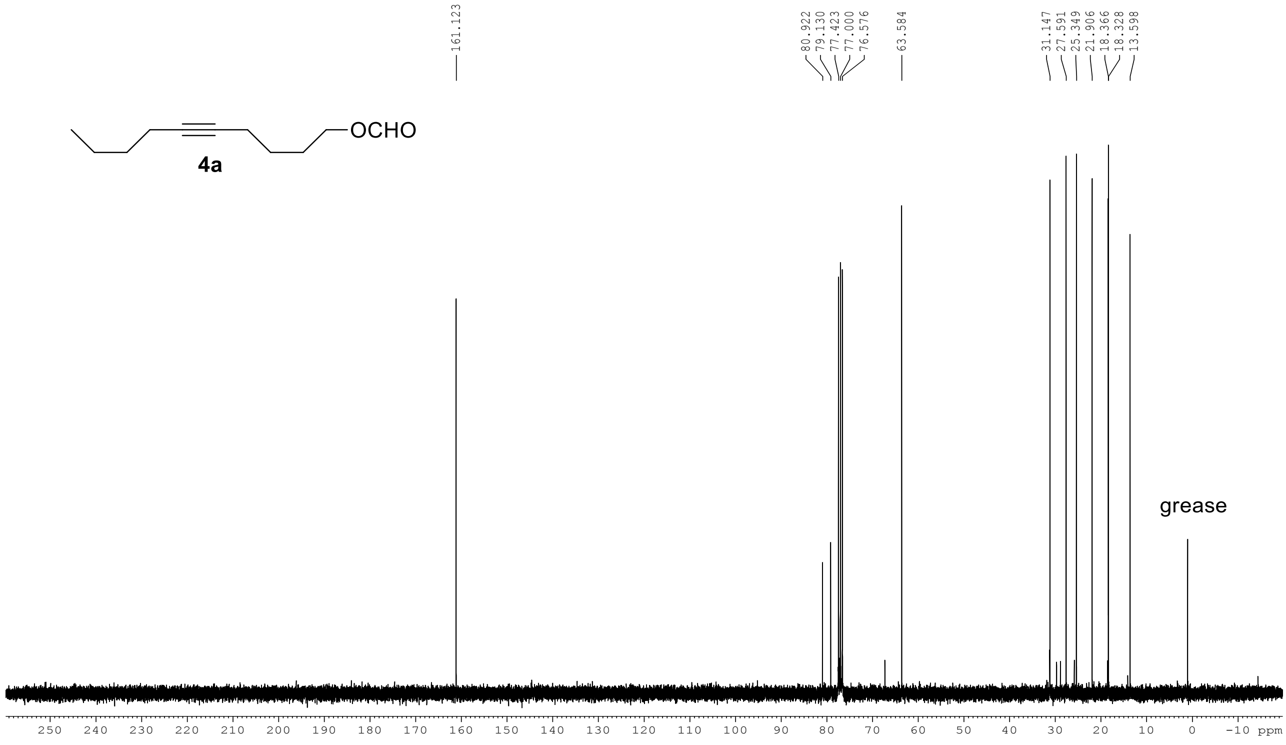
161.123
80.922
79.130
77.423
77.000
76.576
63.584
31.147
27.591
25.349
21.906
18.366
18.328
13.598
OCHO
4a
grease
250 240 230 220 210 200 190 180 170 160 150 140 130 120 110 100 90 80 70 60 50 40 30 20 10 0 -10 ppm

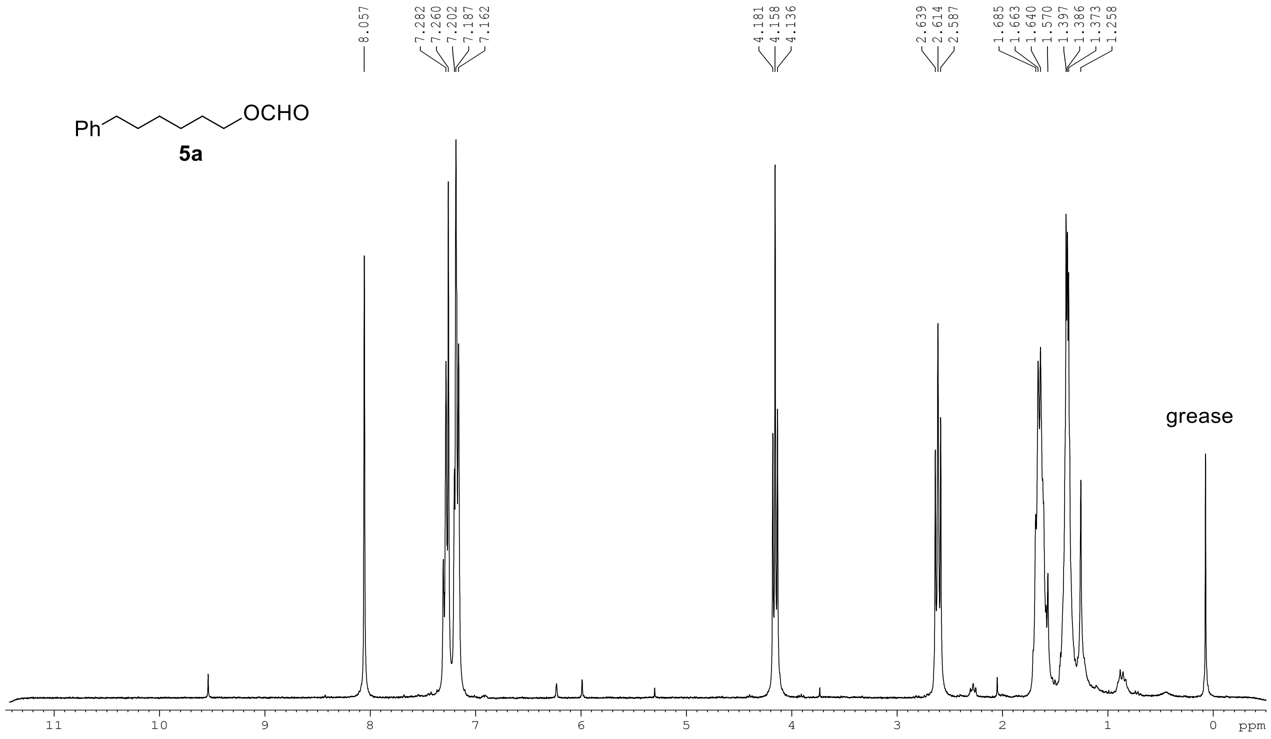
8.057
7.282
7.260
7.202
7.187
7.162
4.181
4.158
4.136
2.639
2.614
2.587
1.685
1.663
1.640
1.570
1.397
1.386
1.373
1.258
Ph OCHO
5a
grease
11 10 9 8 7 6 5 4 3 2 1 0 ppm

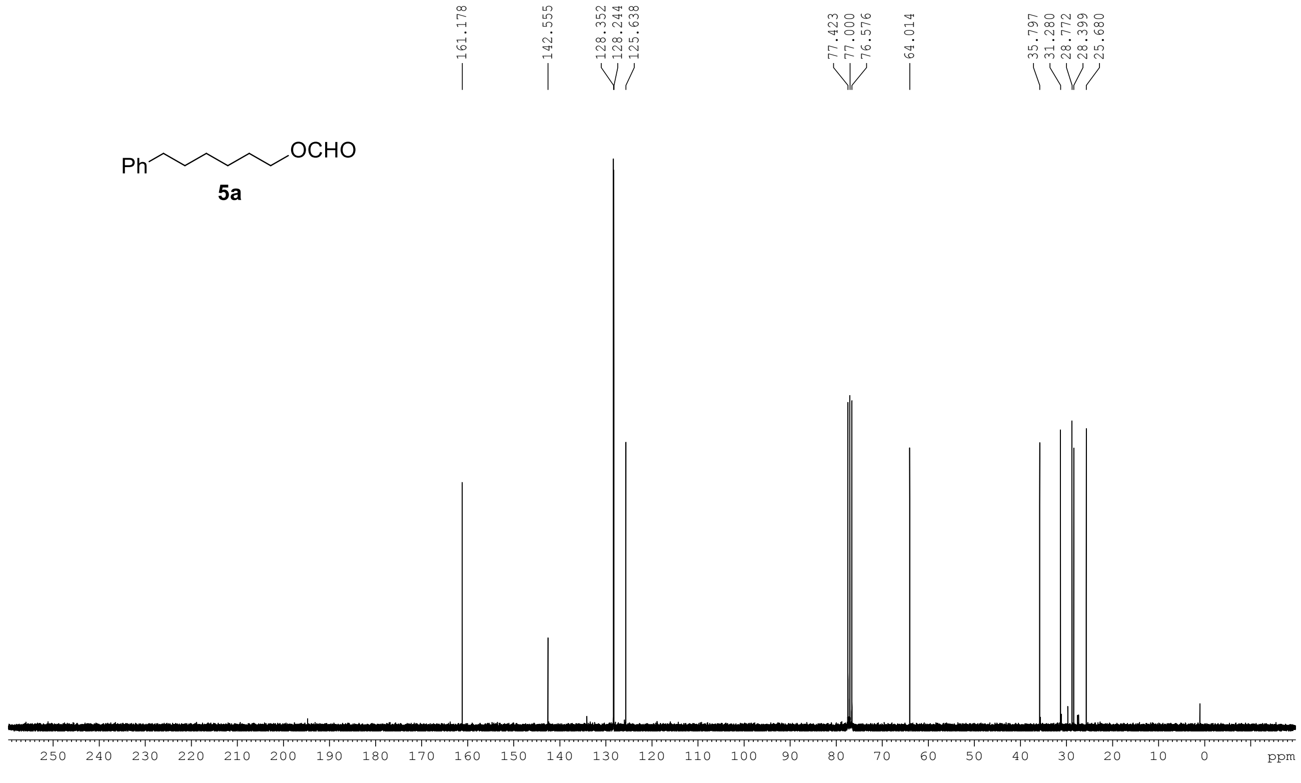
161.178
142.555
128.352
128.244
125.638
77.423
77.000
76.576
64.014
35.797
31.280
28.772
28.399
25.680
Ph OCHO
5a
250 240 230 220 210 200 190 180 170 160 150 140 130 120 110 100 90 80 70 60 50 40 30 20 10 0 ppm

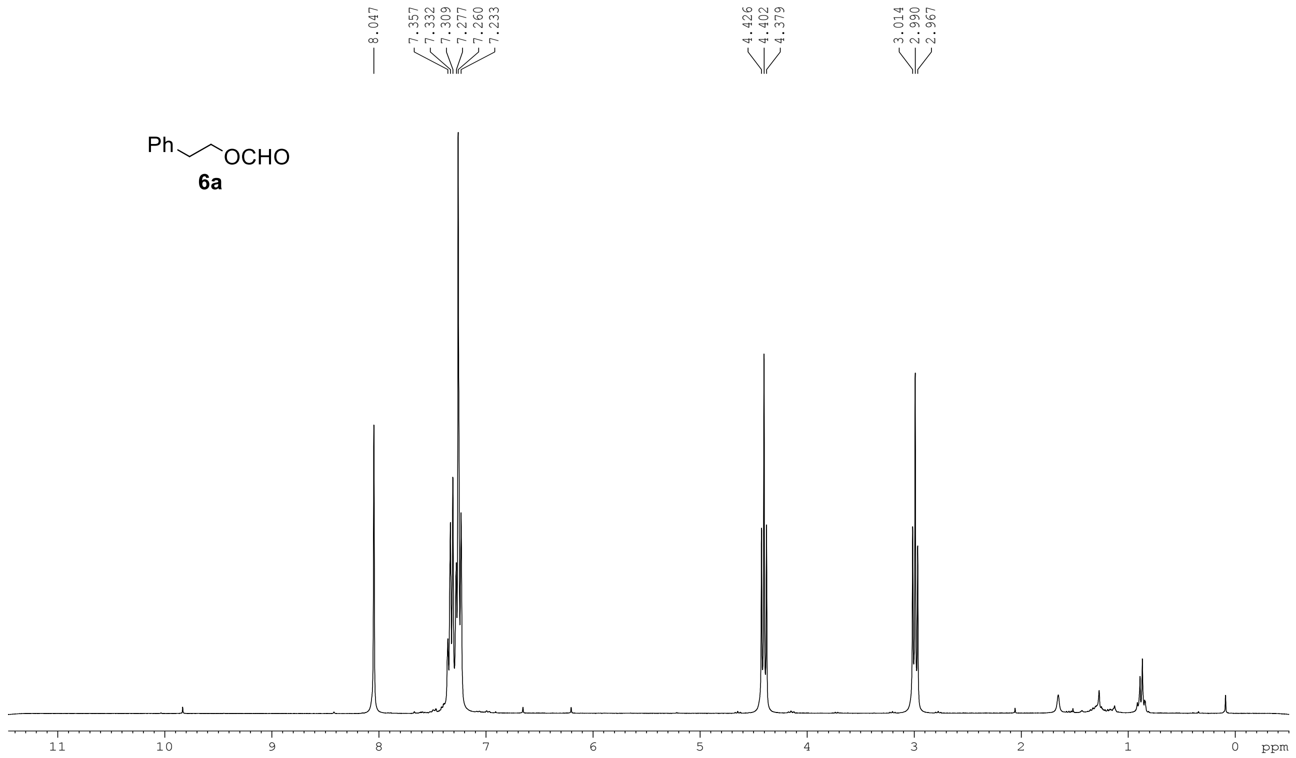
8.047
7.357
7.332
7.309
7.277
7.260
7.233
4.426
4.402
4.379
3.014
2.990
2.967
Ph
OCHO
6a
11 10 9 8 7 6 5 4 3 2 1 0 ppm

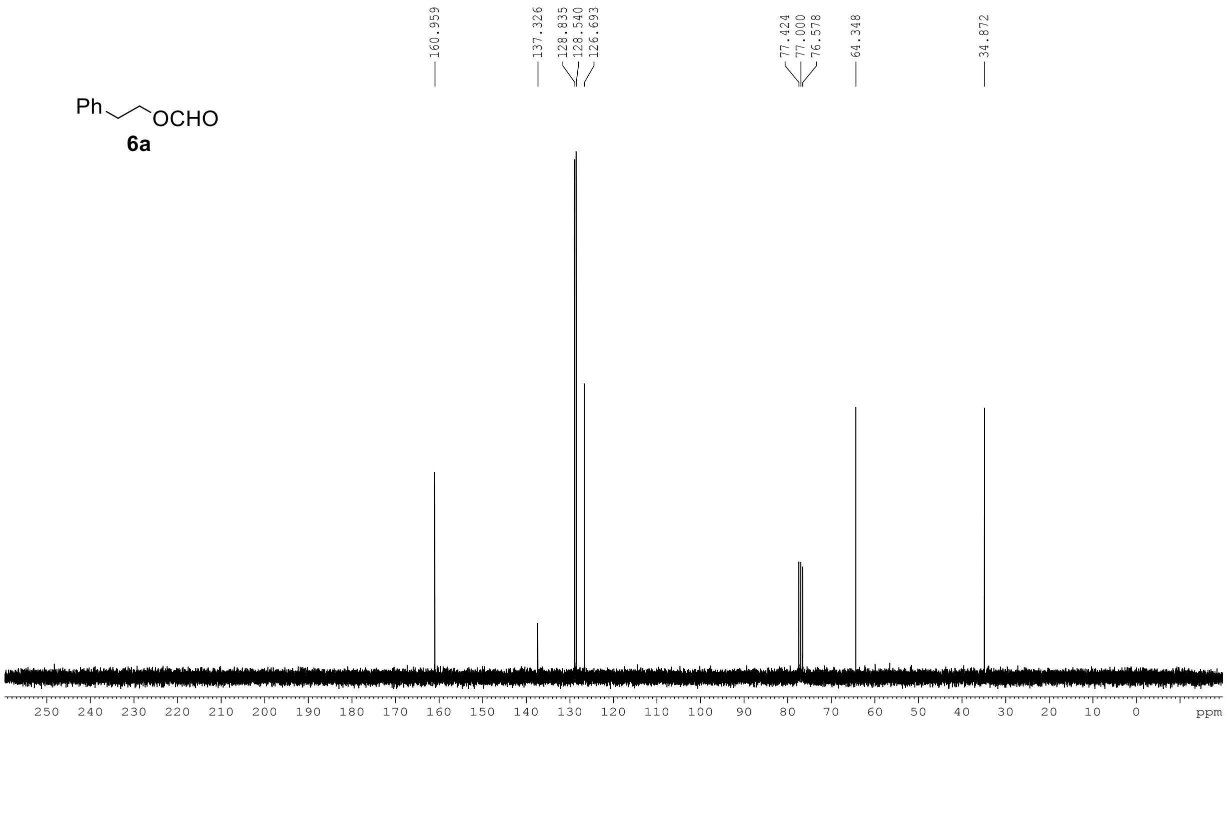
160.959
137.326
128.835
128.540
126.693
77.424
77.000
76.578
64.348
34.872
Ph
OCHO
6a
250 240 230 220 210 200 190 180 170 160 150 140 130 120 110 100 90 80 70 60 50 40 30 20 10 0 ppm

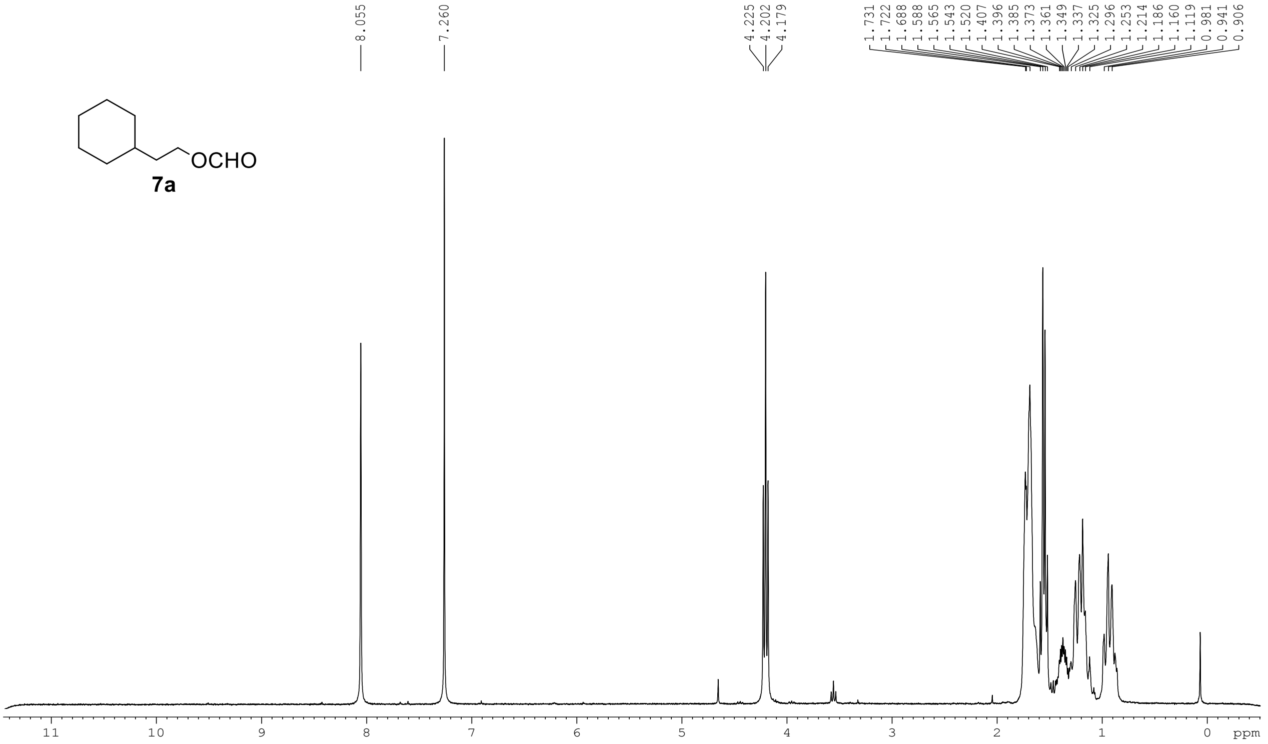

8.055
7.260
4.225
4.202
4.179
1.731
1.722
1.688
1.588
1.565
1.543
1.520
1.407
1.396
1.385
1.373
1.361
1.349
1.337
1.325
1.296
1.253
1.214
1.186
1.160
1.119
0.981
0.941
0.906
OCHO
7a
ppm

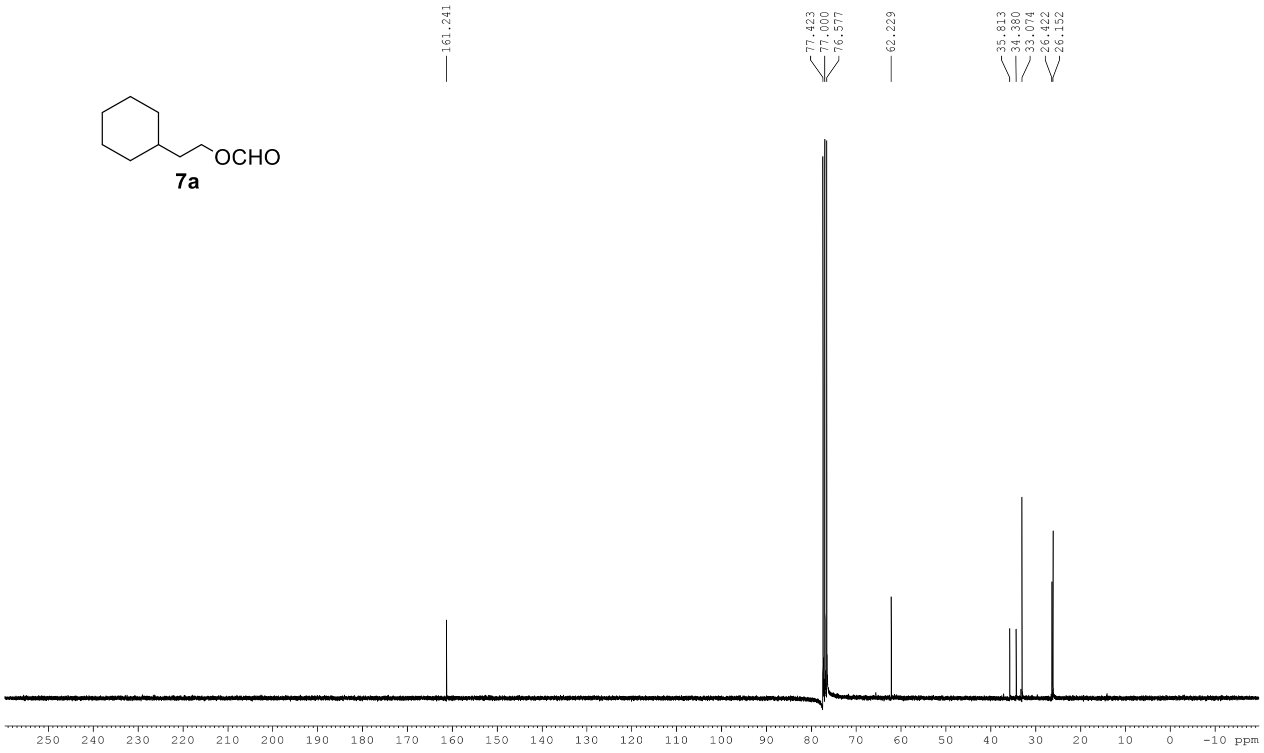

161.241
77.423
77.000
76.577
62.229
35.813
34.380
33.074
26.422
26.152
OCHO
7a
ppm

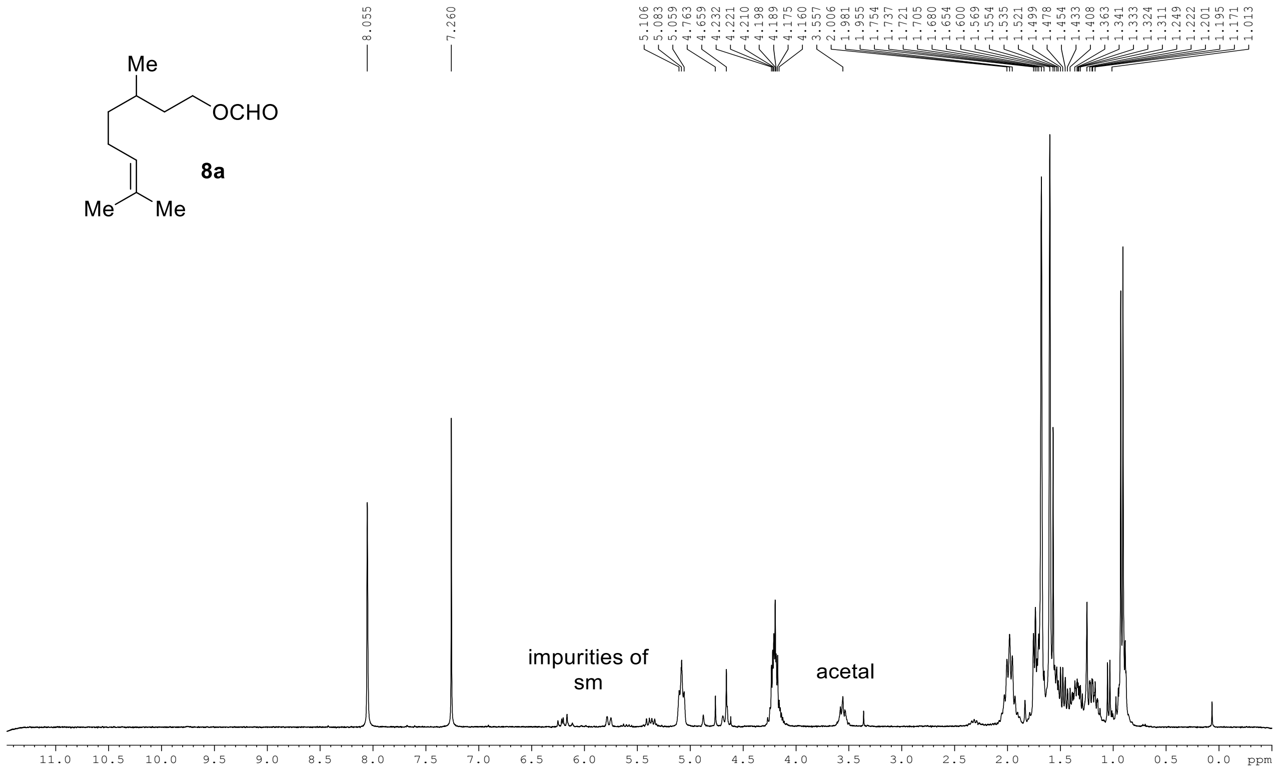
Me
OCHO
8a
Me
Me
8.055
7.260
5.106 5.083 5.059 4.763 4.659 4.232 4.221 4.210 4.198 4.189 4.175 4.160 3.557 2.006 1.981 1.955 1.754 1.737 1.721 1.705 1.680 1.654 1.600 1.569 1.554 1.535 1.521 1.499 1.478 1.454 1.433 1.408 1.363 1.341 1.333 1.324 1.311 1.249 1.222 1.201 1.195 1.171 1.013
impurities of sm
acetal
11.0 10.5 10.0 9.5 9.0 8.5 8.0 7.5 7.0 6.5 6.0 5.5 5.0 4.5 4.0 3.5 3.0 2.5 2.0 1.5 1.0 0.5 0.0 ppm

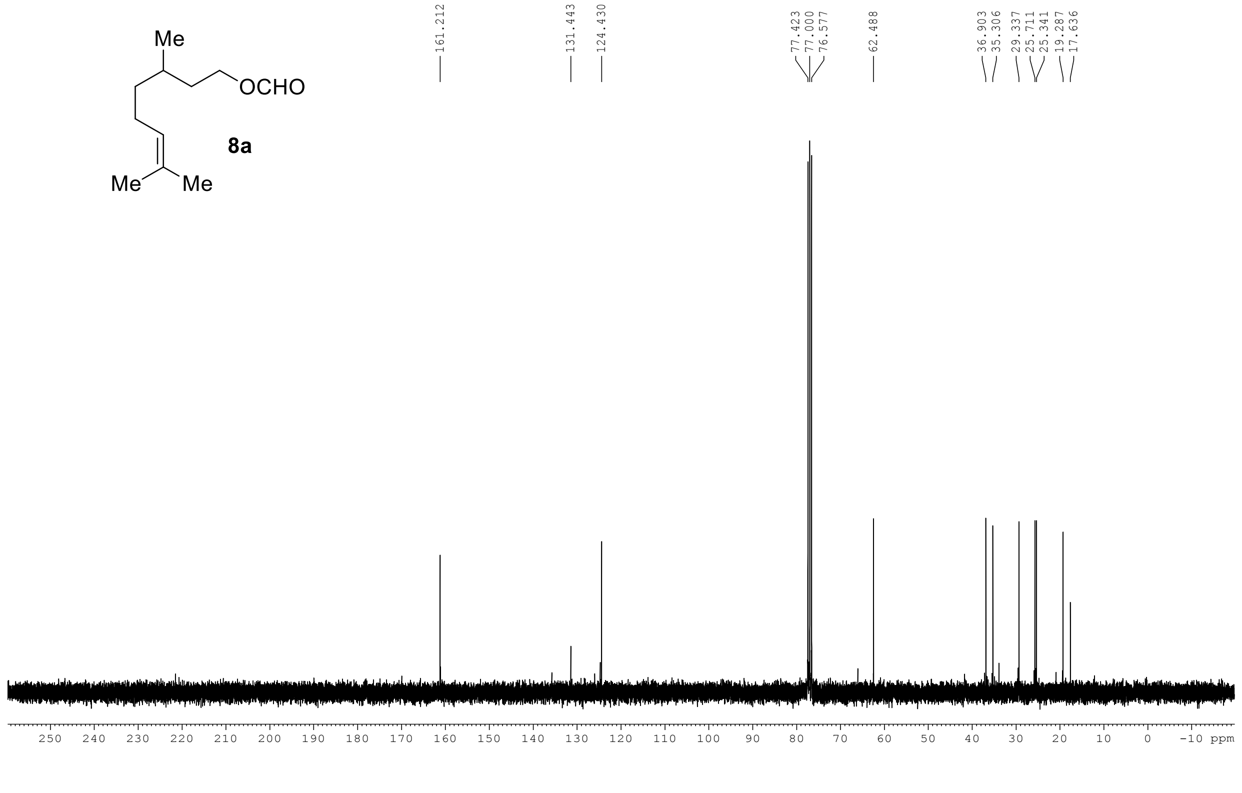
Me
OCHO
8a
Me
Me
161.212
131.443
124.430
77.423
77.000
76.577
62.488
36.903
35.306
29.337
25.711
25.341
19.287
17.636
250 240 230 220 210 200 190 180 170 160 150 140 130 120 110 100 90 80 70 60 50 40 30 20 10 0 -10 ppm

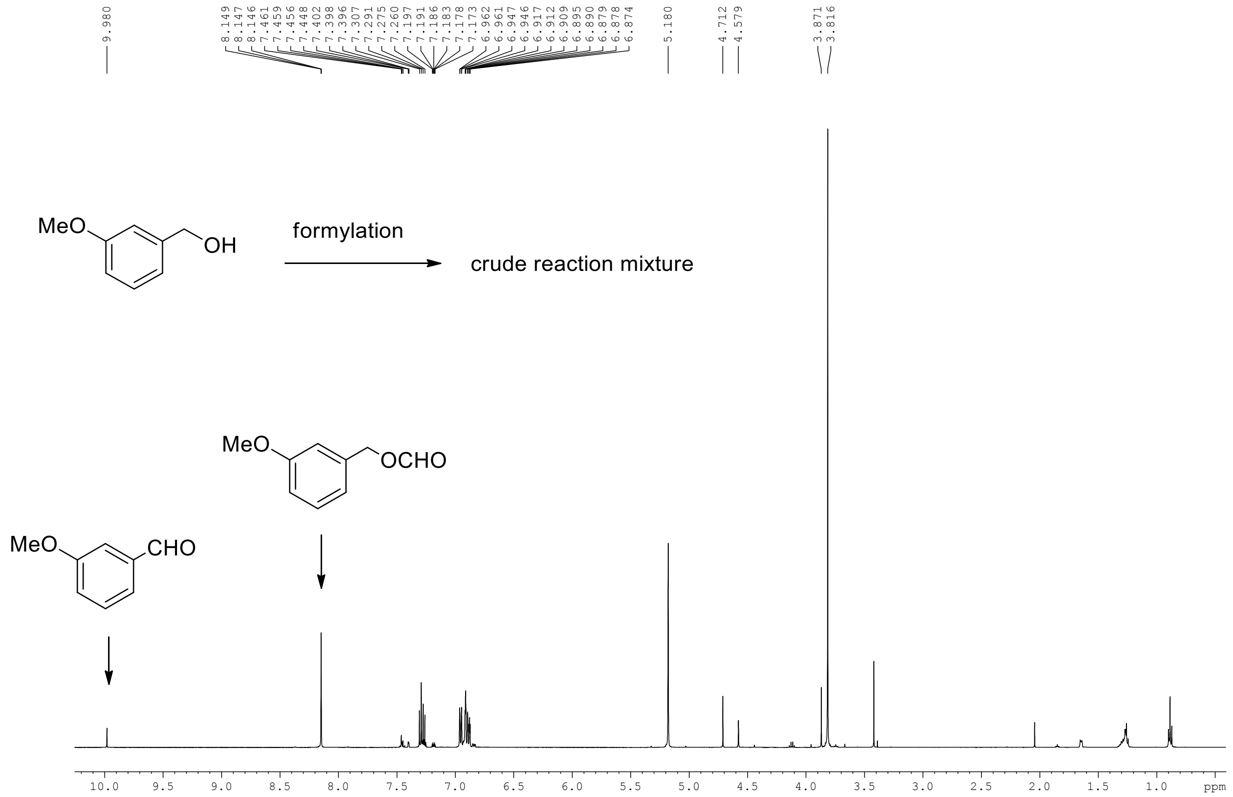

9.980
8.149 8.147 8.146 7.461 7.459 7.456 7.448 7.402 7.398 7.396 7.307 7.291 7.275 7.260 7.197 7.191 7.186 7.183 7.178 7.173 6.962 6.961 6.947 6.946 6.917 6.912 6.909 6.895 6.890 6.879 6.878 6.874
5.180
4.712
4.579
3.871
3.816
MeO
OH
formylation
crude reaction mixture
MeO
OCHO
MeO
CHO
ppm

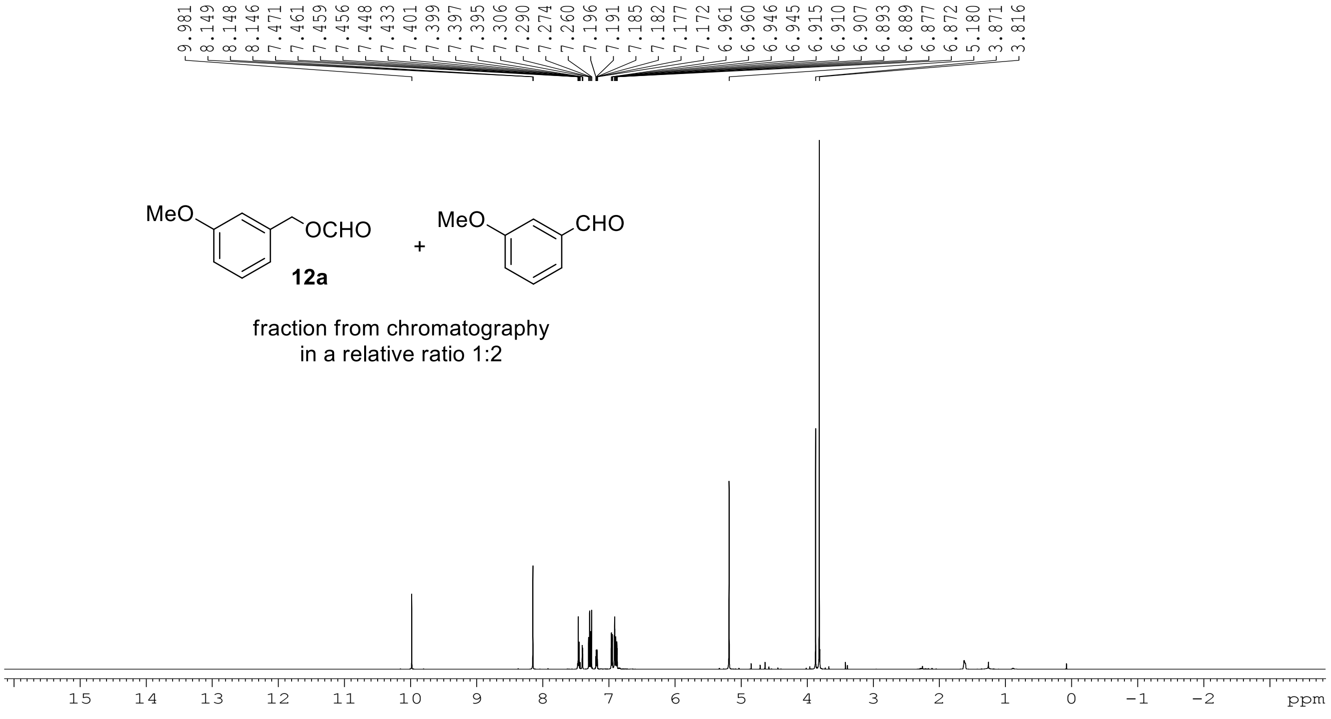

9.981 8.149 8.148 8.146 7.471 7.461 7.459 7.456 7.448 7.433 7.401 7.399 7.397 7.395 7.306 7.290 7.274 7.260 7.196 7.191 7.185 7.182 7.177 7.172 6.961 6.960 6.946 6.945 6.915 6.910 6.907 6.893 6.889 6.877 6.872 5.180 3.871 3.816
MeO
OCHO
12a
+
MeO
CHO
fraction from chromatography
in a relative ratio 1:2
ppm

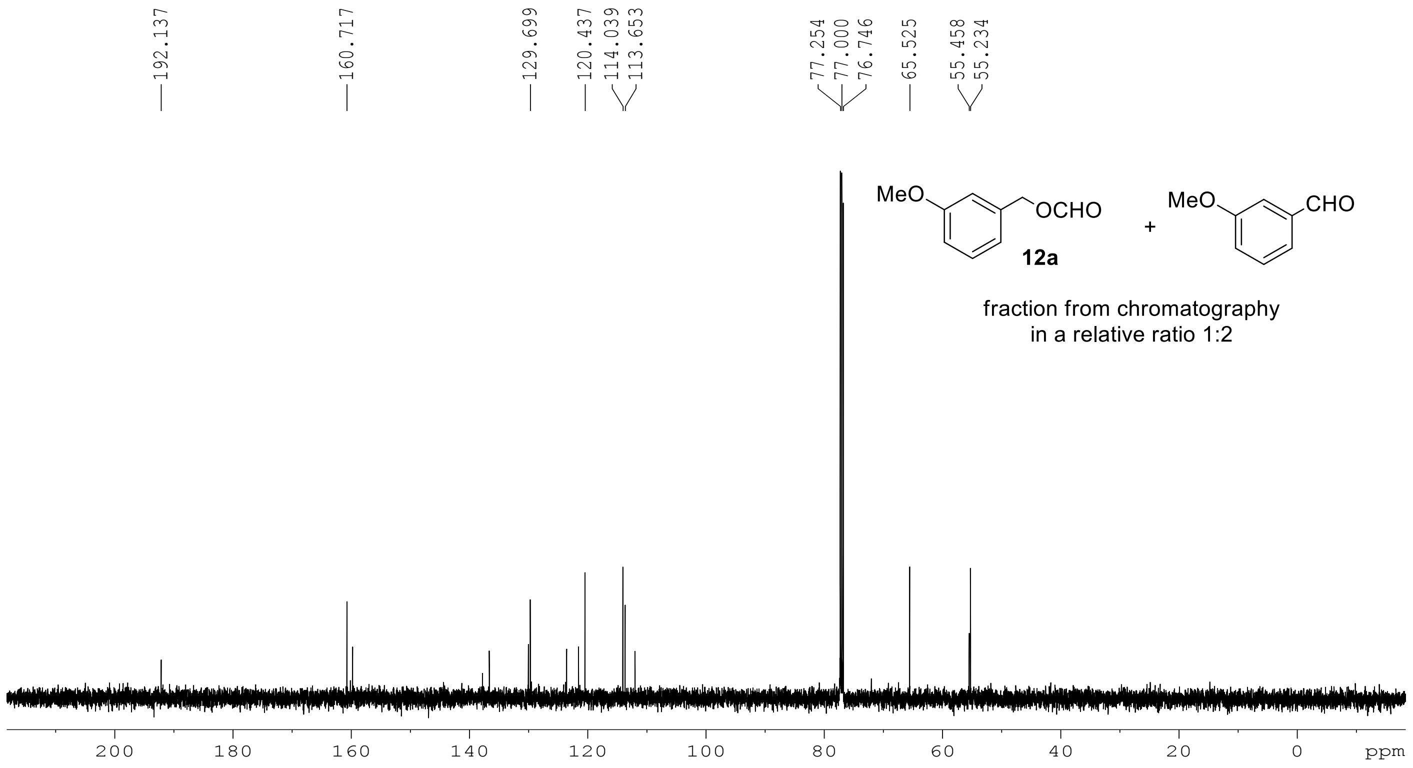

192.137
160.717
129.699
120.437
114.039
113.653
77.254
77.000
76.746
65.525
55.458
55.234
MeO
OCHO
12a
+
MeO
CHO
fraction from chromatography
in a relative ratio 1:2
200
180
160
140
120
100
80
60
40
20
0
ppm

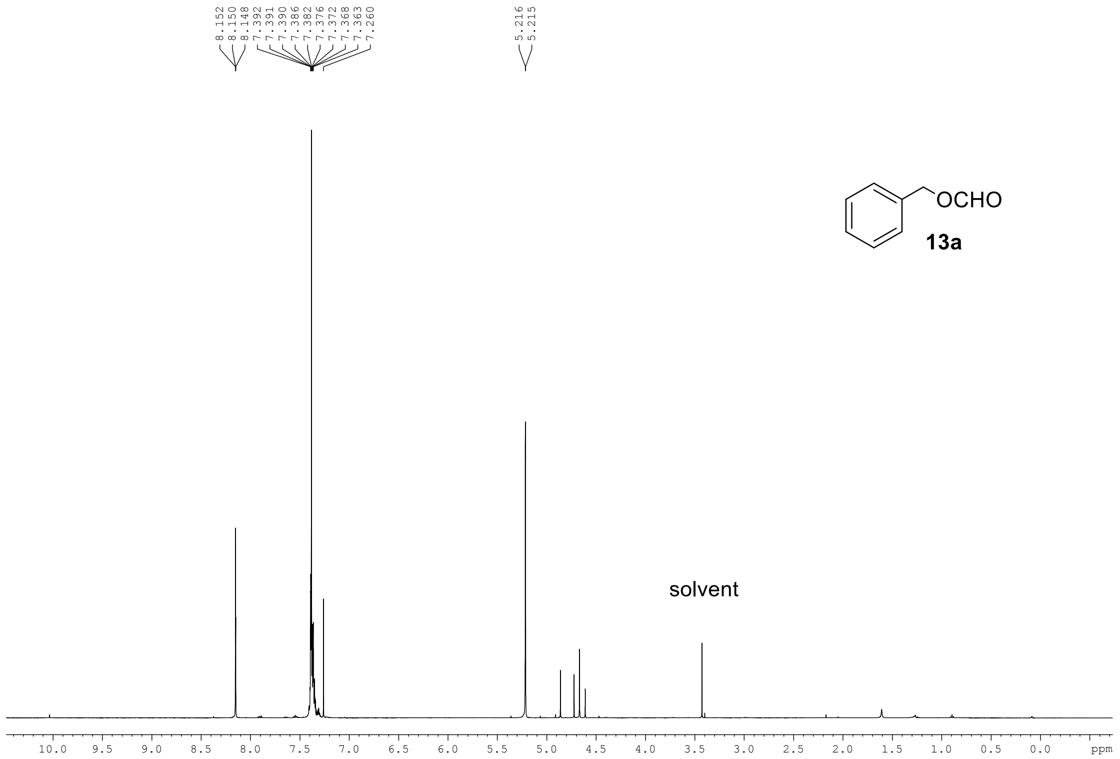

8.152
8.150
8.148
7.392
7.391
7.390
7.386
7.382
7.376
7.372
7.368
7.363
7.260
5.216
5.215
OCHO
13a
solvent
10.0 9.5 9.0 8.5 8.0 7.5 7.0 6.5 6.0 5.5 5.0 4.5 4.0 3.5 3.0 2.5 2.0 1.5 1.0 0.5 0.0 ppm

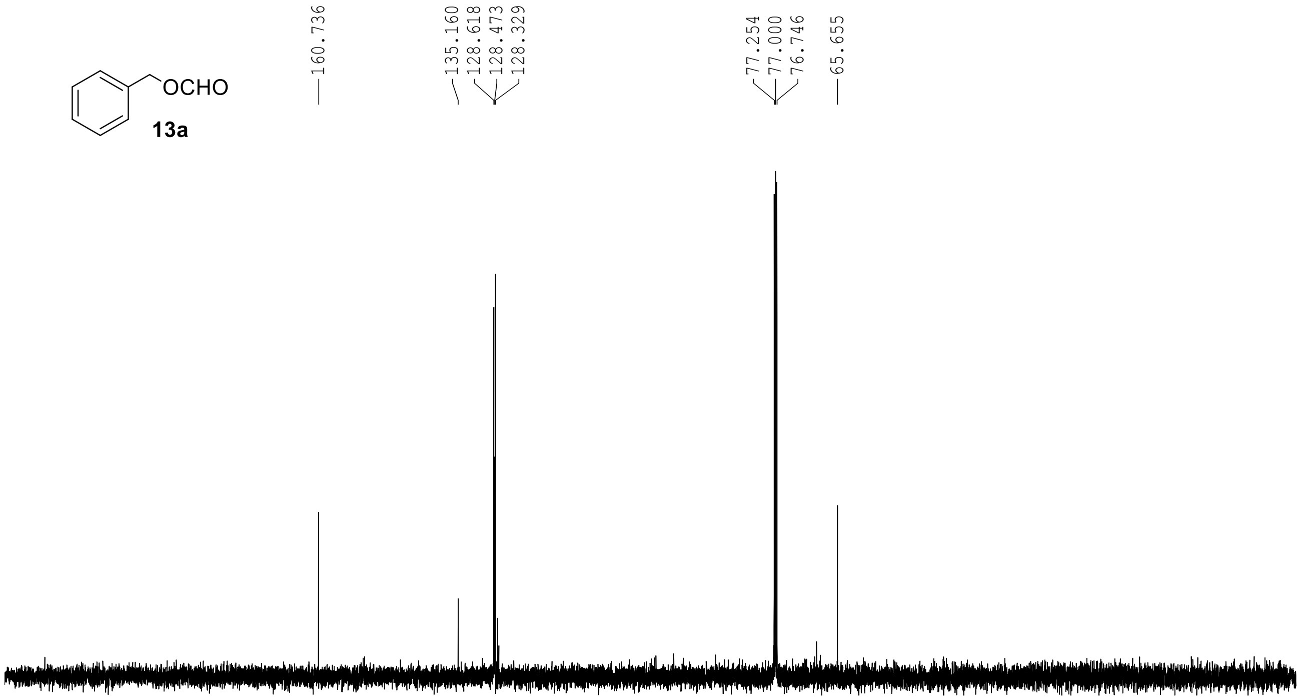

OCHO
13a
160.736
135.160
128.618
128.473
128.329
77.254
77.000
76.746
65.655
200 180 160 140 120 100 80 60 40 20 0 ppm

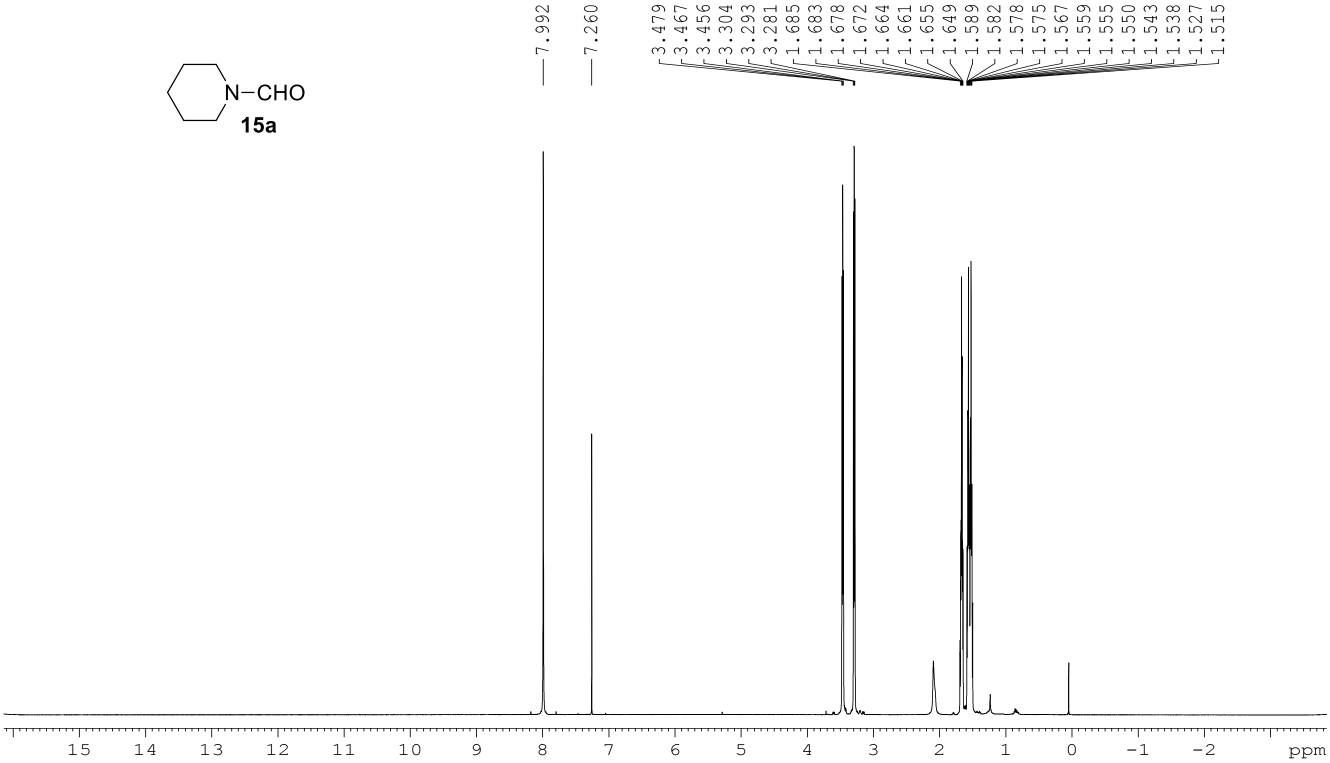
N−CHO
15a
7.992
7.260
3.479
3.467
3.456
3.304
3.293
3.281
1.685
1.683
1.678
1.672
1.664
1.661
1.655
1.649
1.589
1.582
1.578
1.575
1.567
1.559
1.555
1.550
1.543
1.538
1.527
1.515
15 14 13 12 11 10 9 8 7 6 5 4 3 2 1 0 −1 −2 ppm

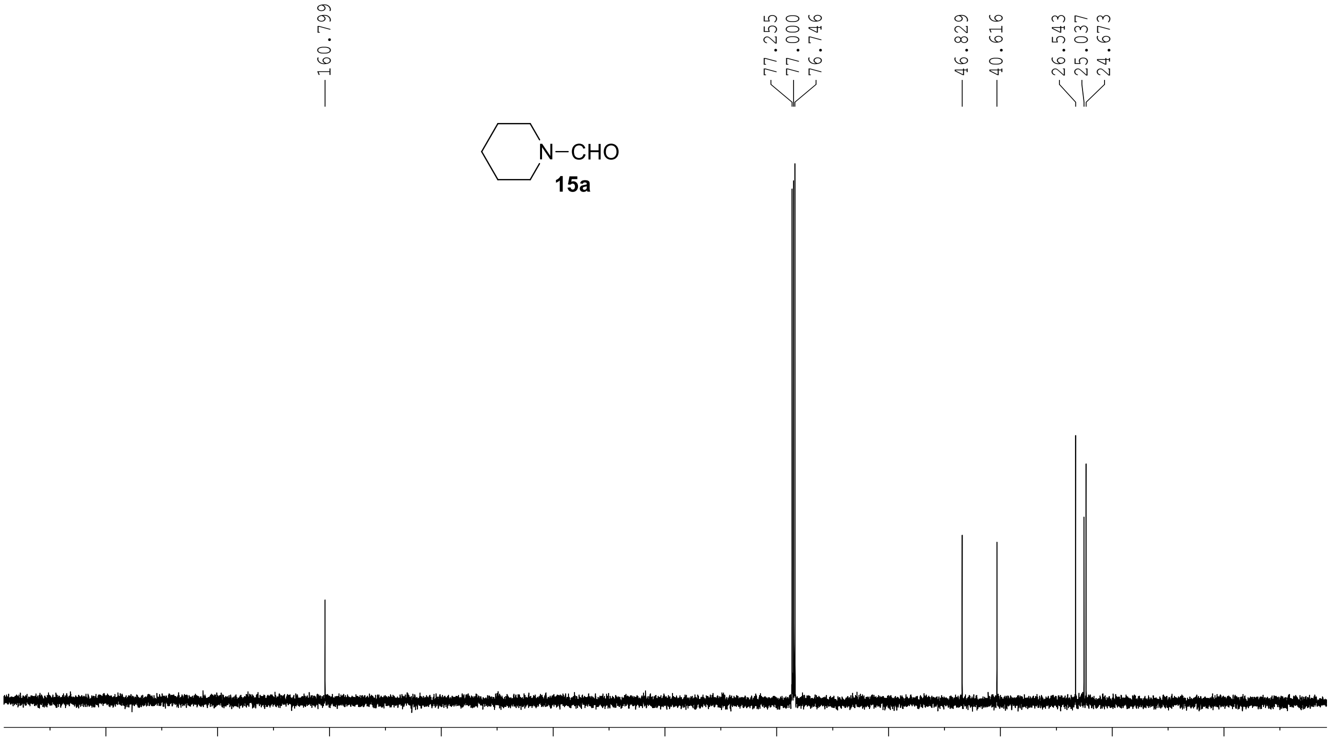
N−CHO
15a
160.799
77.255
77.000
76.746
46.829
40.616
26.543
25.037
24.673
200 180 160 140 120 100 80 60 40 20 0 ppm

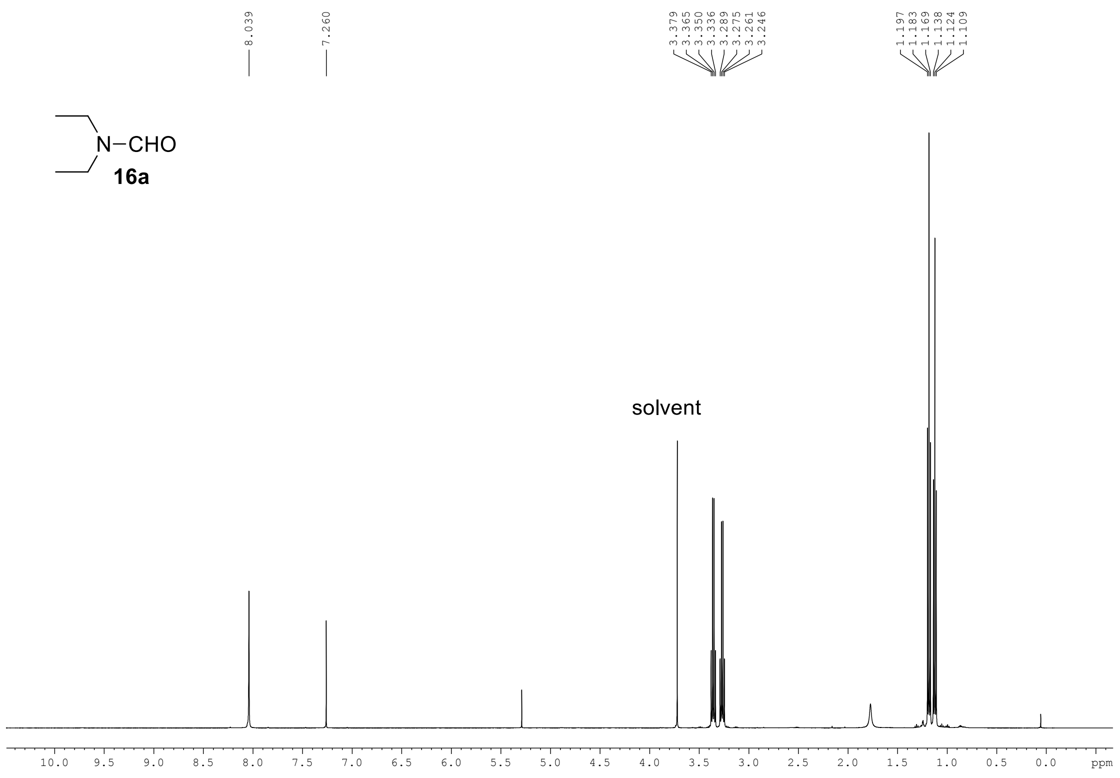
8.039
7.260
3.379
3.365
3.350
3.336
3.289
3.275
3.261
3.246
1.197
1.183
1.169
1.138
1.124
1.109
N−CHO
16a
solvent
10.0 9.5 9.0 8.5 8.0 7.5 7.0 6.5 6.0 5.5 5.0 4.5 4.0 3.5 3.0 2.5 2.0 1.5 1.0 0.5 0.0 ppm

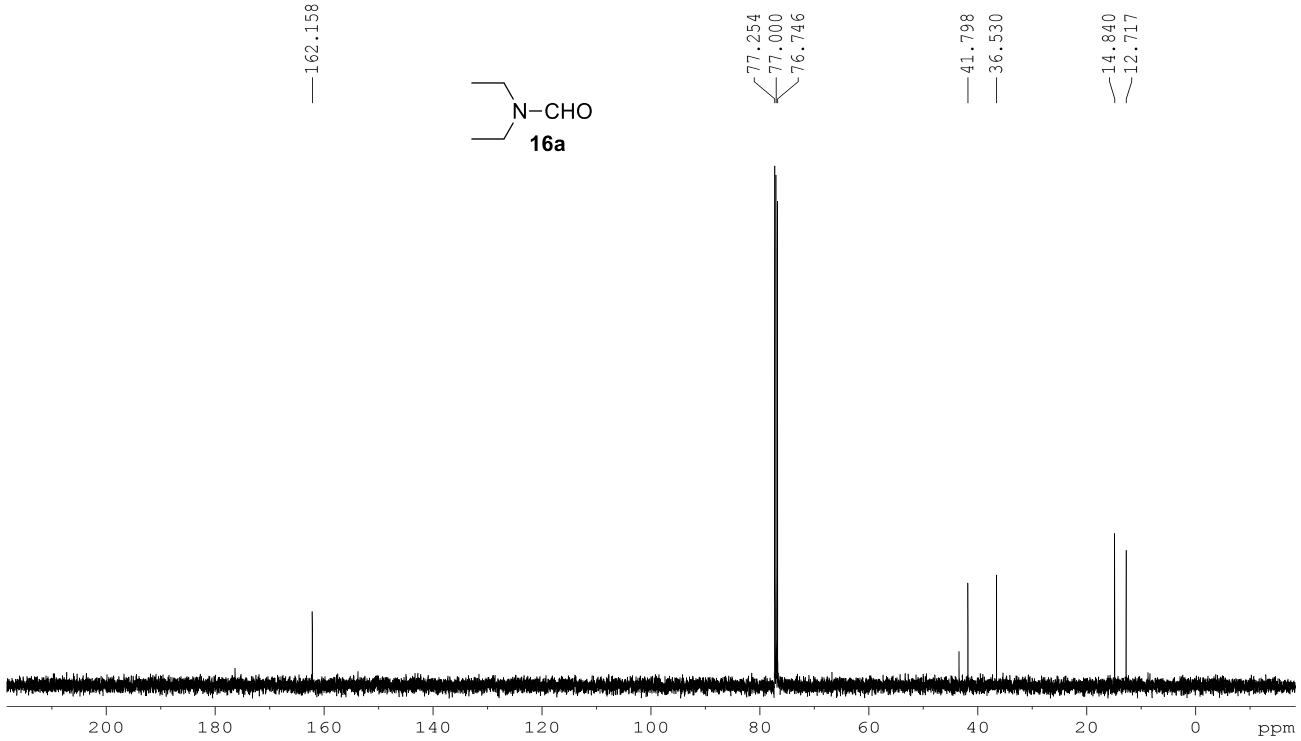
162.158
77.254
77.000
76.746
41.798
36.530
14.840
12.717
N−CHO
16a
200 180 160 140 120 100 80 60 40 20 0 ppm

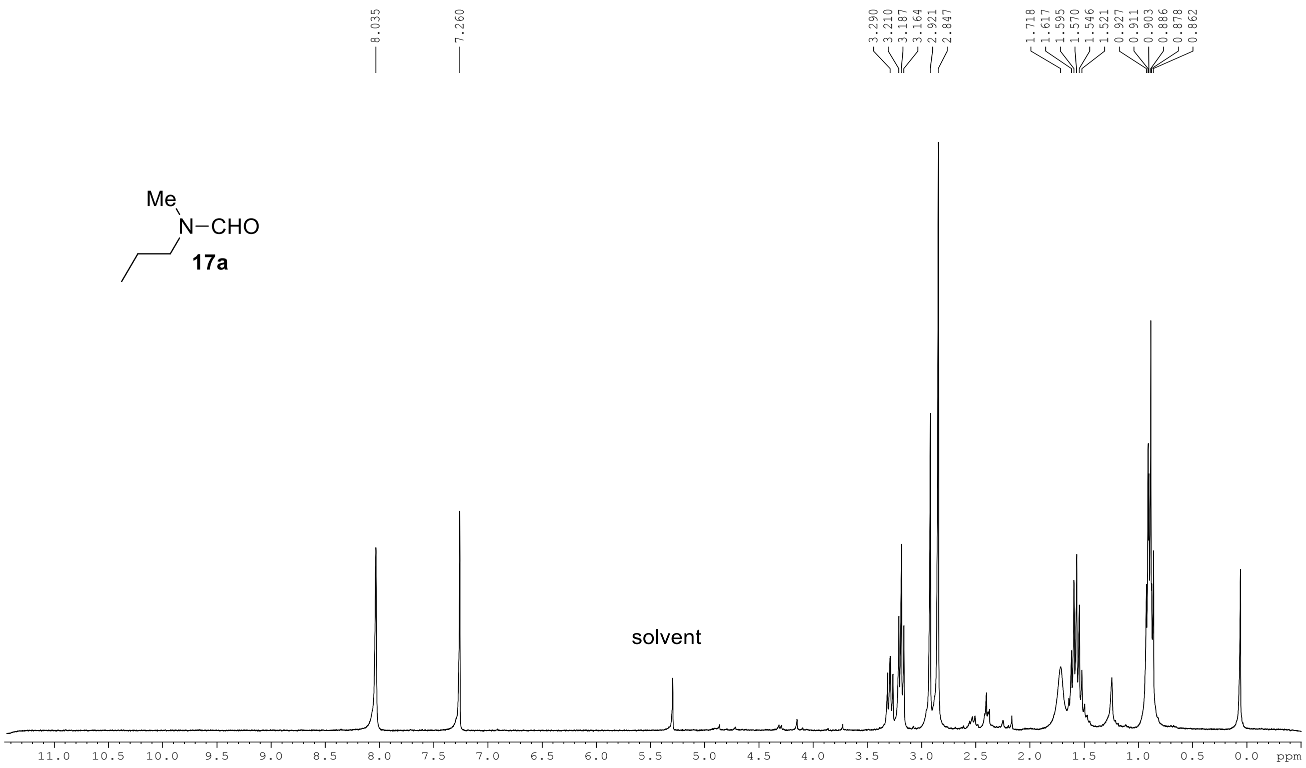
Me
N−CHO
17a
solvent
8.035
7.260
3.290
3.210
3.187
3.164
2.921
2.847
1.718
1.617
1.595
1.570
1.546
1.521
0.927
0.911
0.903
0.886
0.878
0.862
11.0 10.5 10.0 9.5 9.0 8.5 8.0 7.5 7.0 6.5 6.0 5.5 5.0 4.5 4.0 3.5 3.0 2.5 2.0 1.5 1.0 0.5 0.0 ppm

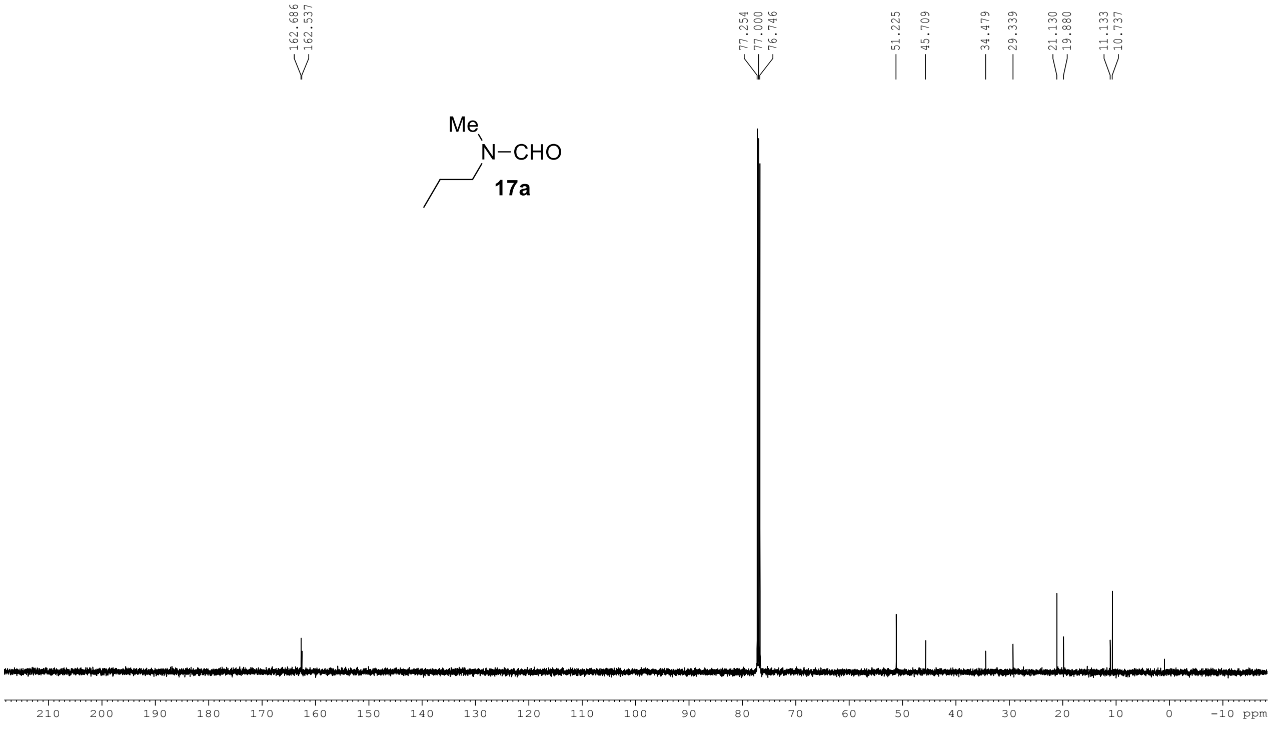
Me
N−CHO
17a
162.686
162.537
77.254
77.000
76.746
51.225
45.709
34.479
29.339
21.130
19.880
11.133
10.737
210 200 190 180 170 160 150 140 130 120 110 100 90 80 70 60 50 40 30 20 10 0 −10 ppm

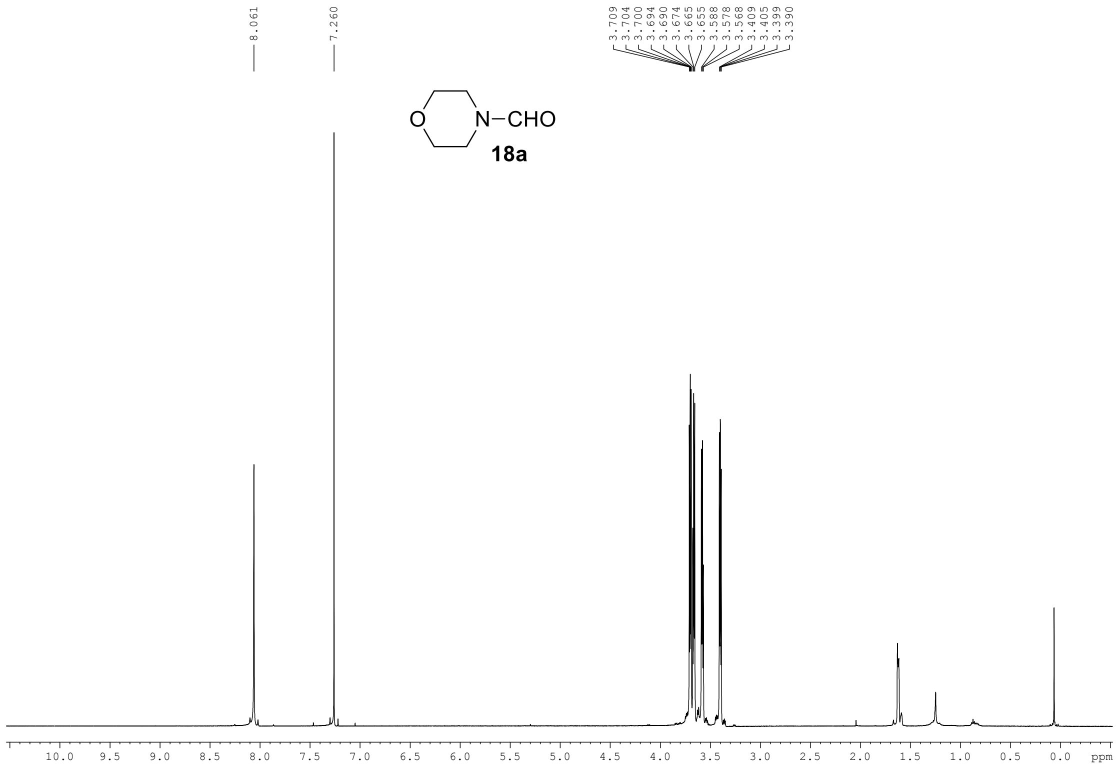
8.061
7.260
3.709
3.704
3.700
3.694
3.690
3.674
3.665
3.655
3.588
3.578
3.568
3.409
3.405
3.399
3.390
N−CHO
18a
ppm

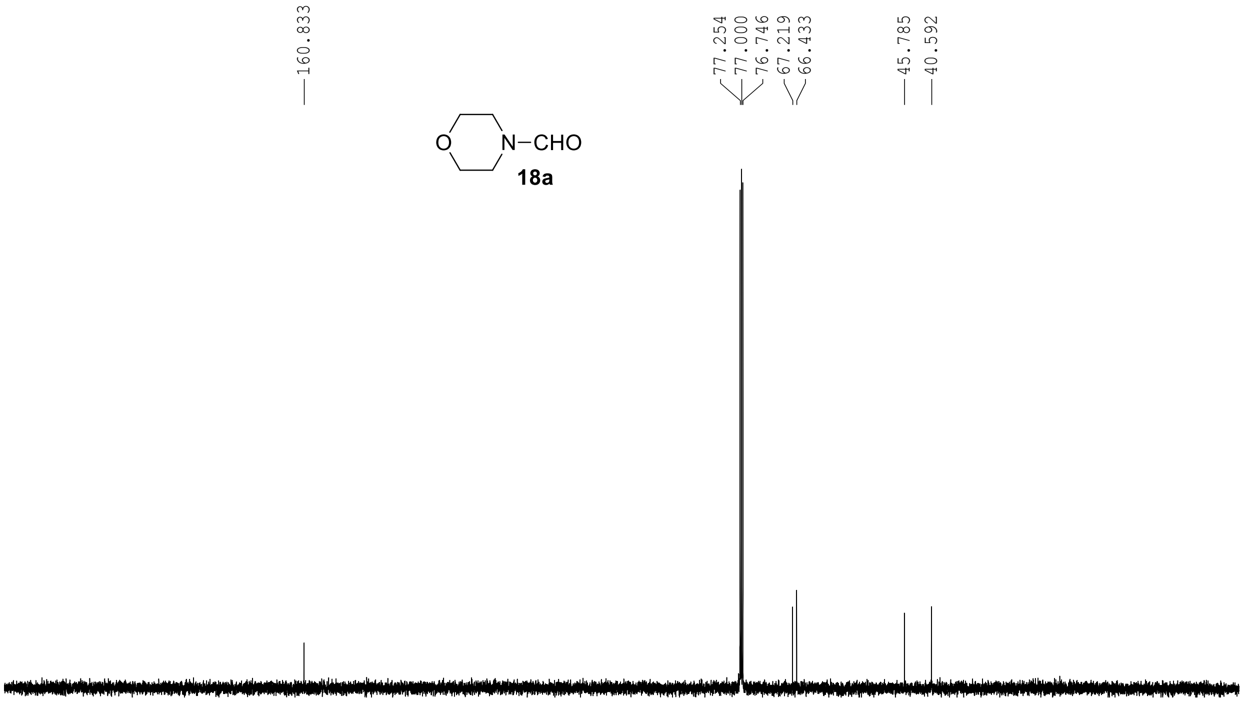
160.833
77.254
77.000
76.746
67.219
66.433
45.785
40.592
N−CHO
18a
ppm

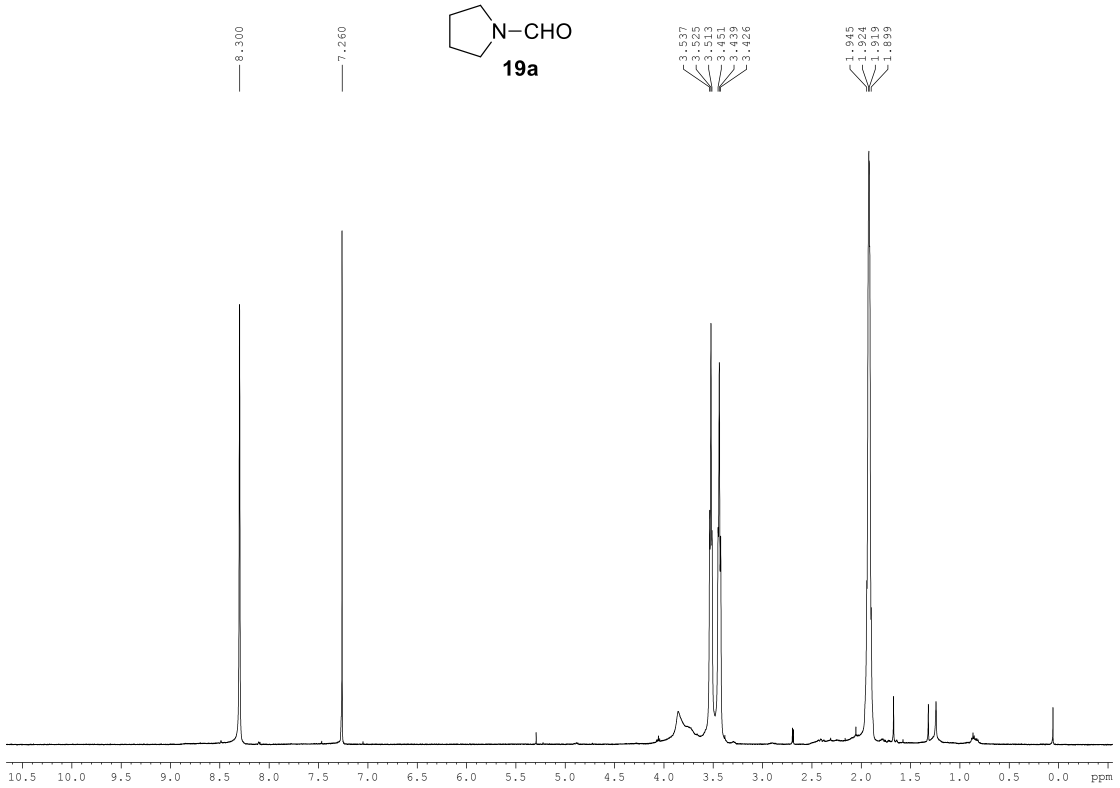
8.300
7.260
N–CHO
19a
3.537
3.525
3.513
3.451
3.439
3.426
1.945
1.924
1.919
1.899
10.5 10.0 9.5 9.0 8.5 8.0 7.5 7.0 6.5 6.0 5.5 5.0 4.5 4.0 3.5 3.0 2.5 2.0 1.5 1.0 0.5 0.0 ppm

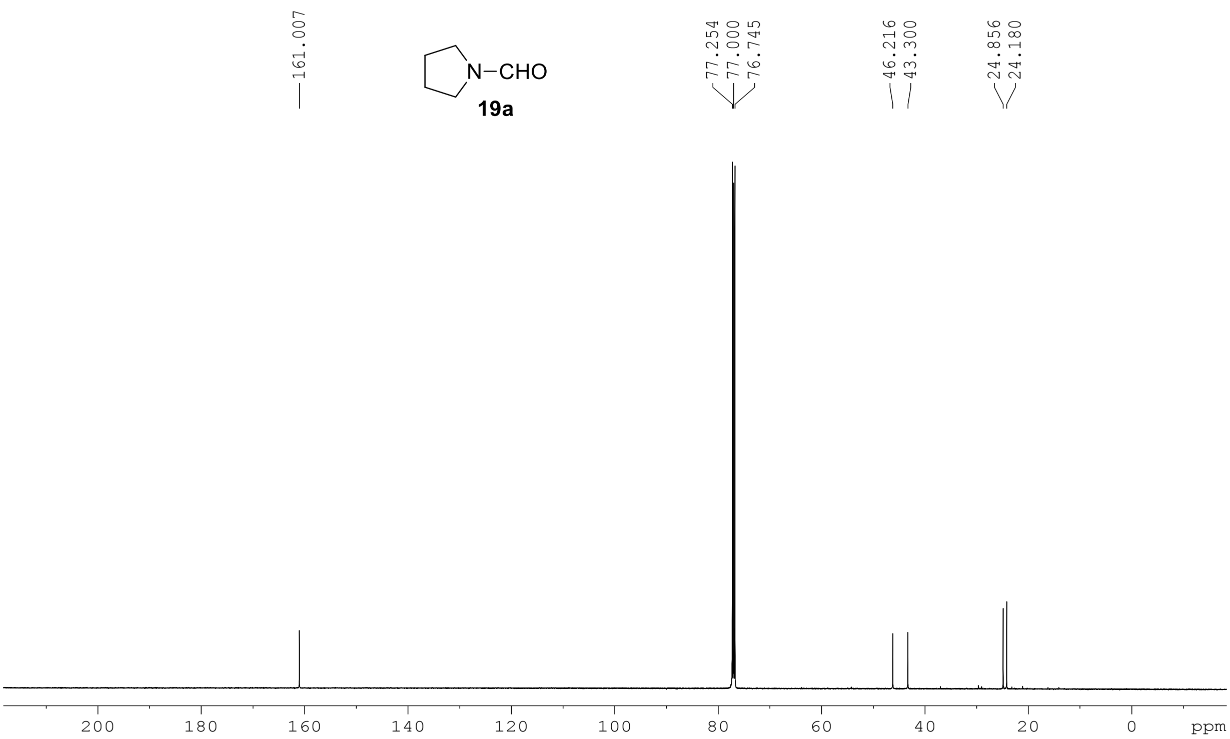
161.007
N–CHO
19a
77.254
77.000
76.745
46.216
43.300
24.856
24.180
200 180 160 140 120 100 80 60 40 20 0 ppm

1

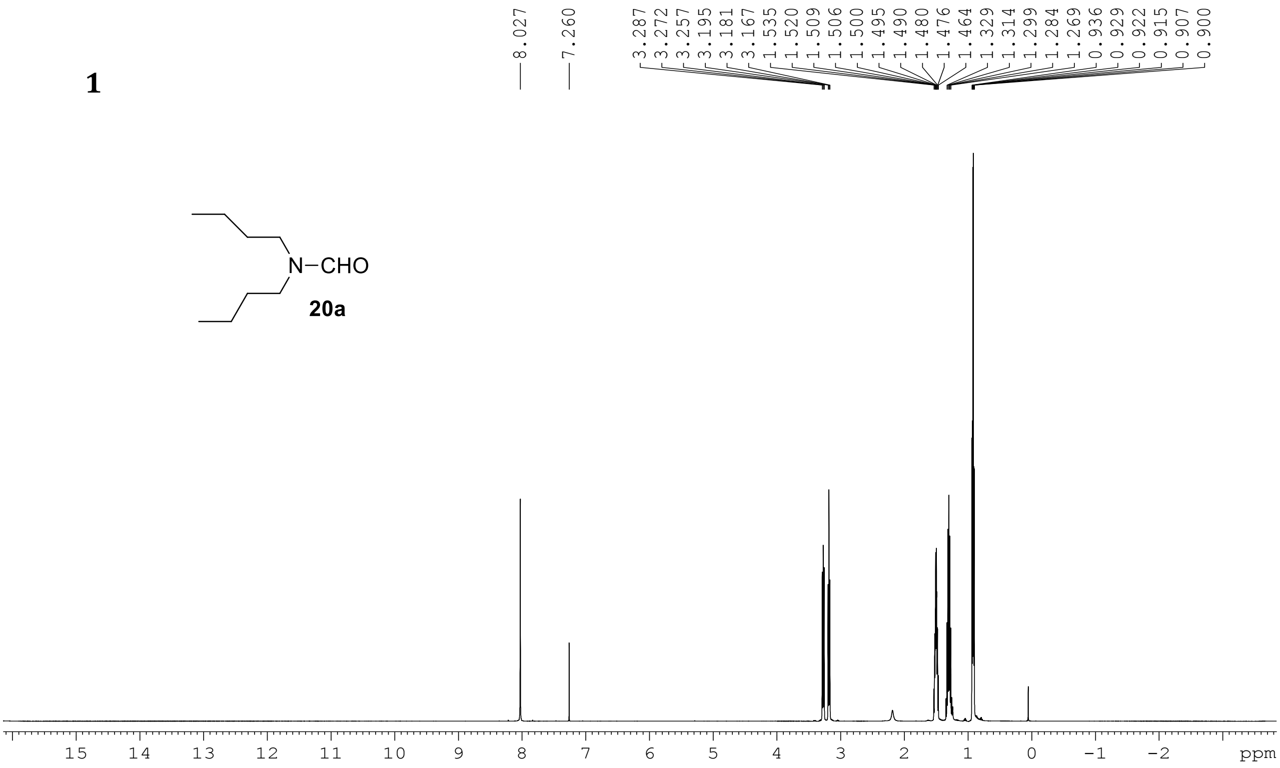
8.027
7.260
3.287
3.272
3.257
3.195
3.181
3.167
1.535
1.520
1.509
1.506
1.500
1.495
1.490
1.480
1.476
1.464
1.329
1.314
1.299
1.284
1.269
0.936
0.929
0.922
0.915
0.907
0.900
N−CHO
20a
15 14 13 12 11 10 9 8 7 6 5 4 3 2 1 0 -1 -2 ppm

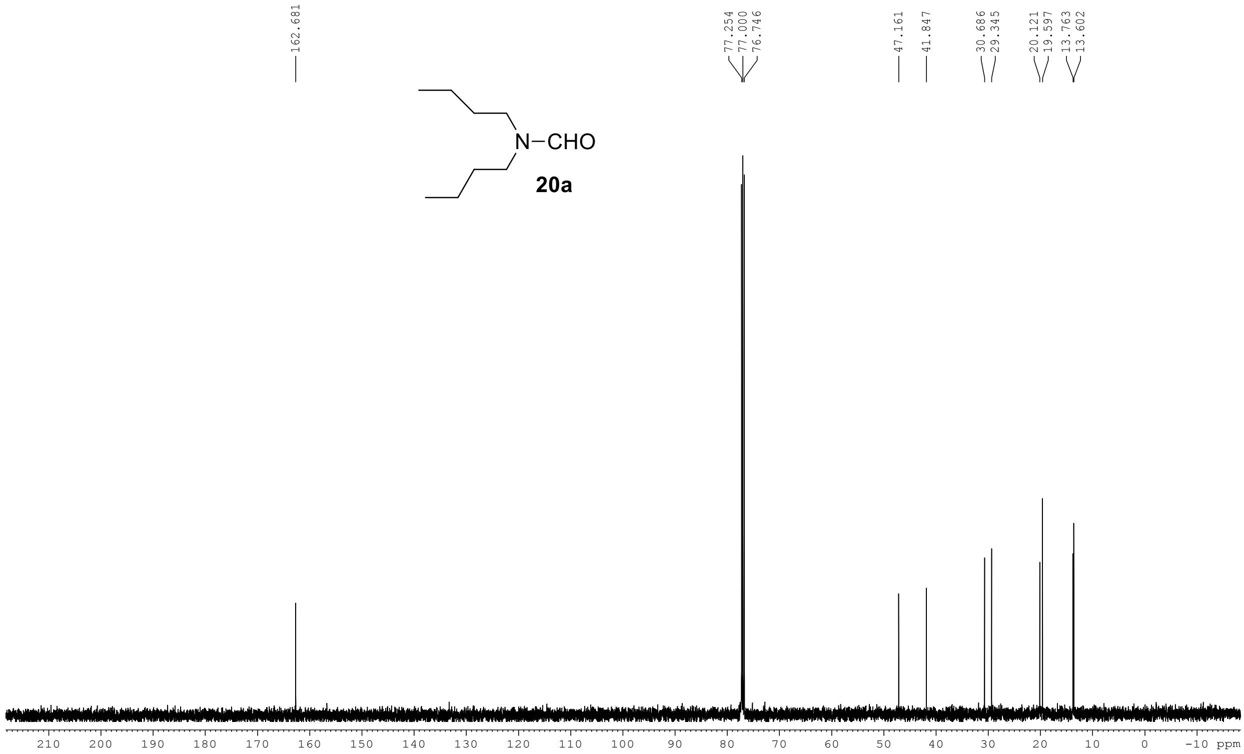
162.681
77.254
77.000
76.746
47.161
41.847
30.686
29.345
20.121
19.597
13.763
13.602
N−CHO
20a
210 200 190 180 170 160 150 140 130 120 110 100 90 80 70 60 50 40 30 20 10 0 -10 ppm

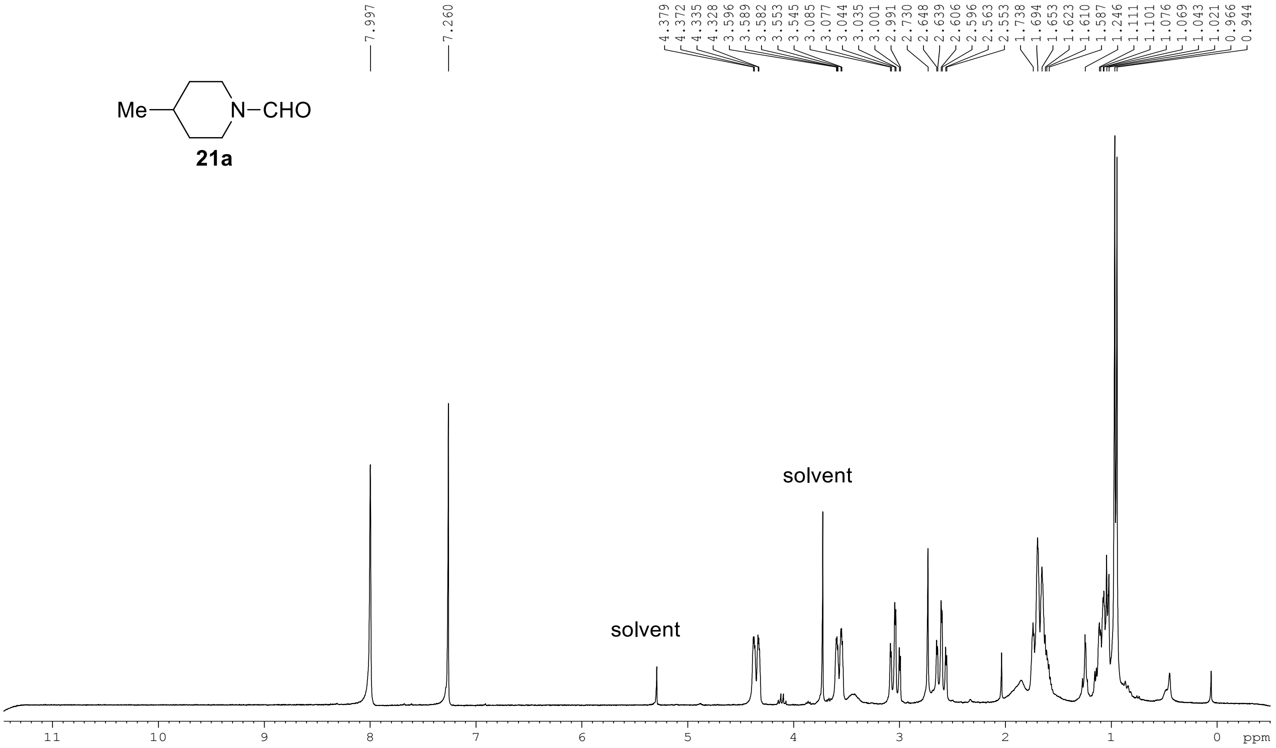
Me
N−CHO
7.997
7.260
4.379 4.372 4.335 4.328 3.596 3.589 3.582 3.553 3.545 3.085 3.077 3.044 3.035 3.001 2.991 2.730 2.648 2.639 2.606 2.596 2.563 2.553 1.738 1.694 1.653 1.623 1.610 1.587 1.246 1.111 1.101 1.076 1.069 1.043 1.021 0.966 0.944
solvent
solvent
11 10 9 8 7 6 5 4 3 2 1 0 ppm

21a

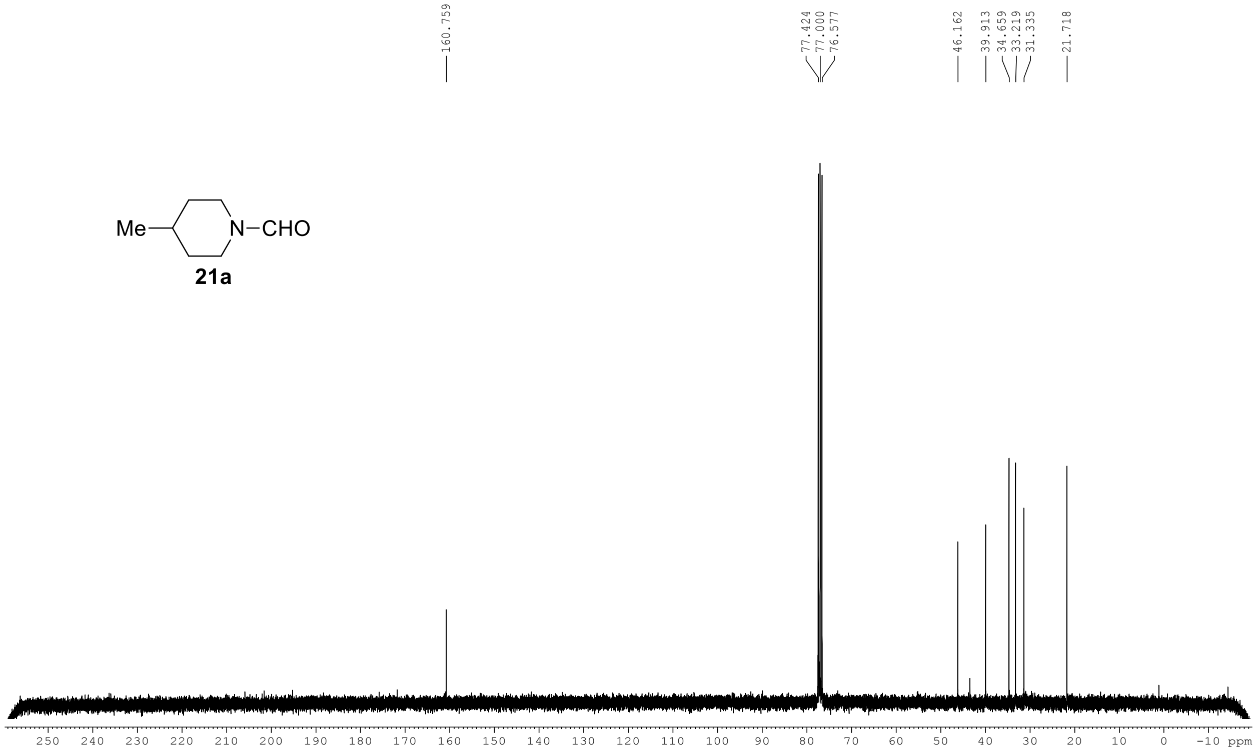
Me
N−CHO
160.759
77.424
77.000
76.577
46.162
39.913
34.659
33.219
31.335
21.718
250 240 230 220 210 200 190 180 170 160 150 140 130 120 110 100 90 80 70 60 50 40 30 20 10 0 -10 ppm

21a
